# Adapting Agriculture Platforms for Nutrition: A Case Study of a Participatory, Video-Based Agricultural Extension Platform in India

**DOI:** 10.1371/journal.pone.0164002

**Published:** 2016-10-13

**Authors:** Suneetha Kadiyala, Emily H. Morgan, Shruthi Cyriac, Amy Margolies, Terry Roopnaraine

**Affiliations:** 1 Department of Population Health, Faculty of Epidemiology and Population Health, London School of Hygiene and Tropical Medicine, London, United Kingdom; 2 Leverhulme Centre for Integrative Research on Agriculture and Health (LCIRAH), London, United Kingdom; 3 Division of Nutritional Sciences, Cornell University, Ithaca, New York, United States of America; 4 St. Johns Research Institute, Bengaluru, Karnataka, India; 5 Department of International Health, Bloomberg School of Public Health, Johns Hopkins University, Baltimore, Maryland, United States of America; 6 Independent consultant, Brasilia, DF, Brazil; University of Illinois at Chicago, UNITED STATES

## Abstract

Successful integration of nutrition interventions into large-scale development programmes from nutrition-relevant sectors, such as agriculture, can address critical underlying determinants of undernutrition and enhance the coverage and effectiveness of on-going nutrition-specific activities. However, evidence on how this can be done is limited. This study examines the feasibility of delivering maternal, infant, and young child nutrition behaviour change communication through an innovative agricultural extension programme serving nutritionally vulnerable groups in rural India. The existing agriculture programme involves participatory production of low-cost videos promoting best practices and broad dissemination through village-level women’s self-help groups. For the nutrition intervention, 10 videos promoting specific maternal, infant, and young child nutrition practices were produced and disseminated in 30 villages. A range of methods was used to collect data, including in-depth interviews with project staff, frontline health workers, and self-help group members and their families; structured observations of mediated video dissemination sessions; nutrition knowledge tests with project staff and self-help group members; and a social network questionnaire to assess diffusion of promoted nutrition messages. We found the nutrition intervention to be well-received by rural communities and viewed as complementary to existing frontline health services. However, compared to agriculture, nutrition content required more time, creativity, and technical support to develop and deliver. Experimentation with promoted nutrition behaviours was high, but sharing of information from the videos with non-viewers was limited. Key lessons learned include the benefits of and need for collaboration with existing health services; continued technical support for implementing partners; engagement with local cultural norms and beliefs; empowerment of women’s group members to champion nutrition; and enhancement of message diffusion mechanisms to reach pregnant women and mothers of young children at scale. Understanding the experience of developing and delivering this intervention will benefit the design of new nutrition interventions which seek to leverage agriculture platforms.

## Introduction

Maternal and child undernutrition remains a leading global public health challenge with short-, medium-, and long-term consequences [1,2]. Despite a substantial increase in commitment to addressing the problem in recent years [3,4], an estimated 3.1 million children under five still die annually of undernutrition and, as of 2011, 165 million children were stunted (low height-for-age) [1]. The burden of maternal and child undernutrition in India is far greater than any other country; the last National Family Health Survey (NFHS) in 2005–06 showed that over a third of women of reproductive age were underweight (BMI<18.5 kg/m^2^) and over half were anaemic [5]. Recent preliminary reports from the Rapid Survey on Children 2013–14 show encouraging trends: between 2005–06 and 2013–14, the prevalence of underweight children under five fell from 43.5% to 30.7%, and the prevalence of child stunting fell from 47.9% to 38.8% [6,7].

A woman’s nutritional status at the time of conception and during pregnancy, along with a child’s nutritional status in the first two years, are essential for healthy growth and development [1]. Research has identified several determinants of poor infant and young child nutrition, such as inadequate dietary intake, frequent infection, women’s disempowerment, lack of health services, poor water and sanitation, and underlying cultural, social, and economic factors [1,8]. It has been calculated that implementing a set of ten proven nutrition-specific interventions (i.e. those that address immediate determinants of maternal, infant, and young child nutrition (MIYCN), such a micronutrient supplementation, optimal child feeding practices, and management of severe acute malnutrition) at 90% coverage could reduce deaths and stunting prevalence among children under five by 15% and 20% respectively [9]. However, coverage of these interventions remains limited, especially in sub-Saharan Africa and South Asia, where need is the highest [6]. In India, the last NFHS revealed coverage of 12 essential child nutrition interventions to be under 50%, with particularly low coverage for interventions related to dietary adequacy and hygiene practices [10].

It is now well recognized that acceleration of progress in MIYCN will require multisectoral action that entails coupling effective nutrition-specific interventions with nutrition-sensitive programmes (i.e. those that address the underlying causes of undernutrition) [11]. There is an increasing international interest in understanding how large-scale programmes from nutrition-relevant sectors such as agriculture can address critical underlying determinants of nutrition and simultaneously enhance the coverage and effectiveness of nutrition-specific interventions [11–13]. In India and other parts of South Asia, the very low status of women relative to men has been identified as an important root cause of persistent undernutrition. In fact, empirical analyses of data from 1970 to 2012 indicate that if gender disparities in South Asia were fully addressed, the prevalence of stunting in the region would decline by 10 percentage points [8]. In rural India, agriculture remains the largest source of employment and there is evidence that Indian women in agriculture work extremely hard, long hours, with adverse effects for their own nutrition and that of their children [14]. Given the key role of gender in mediating the pathways between agriculture and MIYCN, the need to empower women in agriculture and nutrition has been highlighted [11].

‘Making agriculture work for nutrition’ is now a top policy priority. The question is how can it be done? This paper contributes to this line of inquiry.

Agricultural extension and advisory services are an integral part of most programmes to improve farmers’ productivity. These services involve knowledge transfer and information flows between researchers and rural communities with the aim of enhancing rural development [15]. In low and middle income countries, they most often entail an extension worker traveling from door-to-door in villages and interacting with a select number of individuals, usually men with larger farms [16,17]. Despite their wide use, extension services have been criticised for problems of scale, accountability, and financial sustainability [15]. Studies have shown that many farmers have little access to extension services and, even when provided access, may be slow to adopt extension agents' advice if they do not possess location-specific knowledge. Additionally, many extension workers visit infrequently or erratically, and critical information does not reach farmers with the lowest yields, many of whom are the poorest and are women [18]. In response, agricultural extension systems in India and other countries are evolving towards pluralistic, decentralized, participatory, and demand-driven approaches to improve coverage and relevance [19,20].

One promising innovation is the use of information and communications technology (ICT) to strengthen the delivery and uptake of extension services even in resource-poor settings [21,22]. Globally, experimentation with ICTs to promote health- and nutrition-related behaviour change is also gaining momentum. Numerous studies support the effectiveness of video-based behaviour change communication (BCC) in modifying health behaviours; however, the majority of interventions come from wealthy countries, particularly the United States [23]. The use of video as a medium for BCC has several possible advantages for low resource settings, including low cost [24], accessibility by illiterate or semi-literate populations [25,26], standardization of the information communicated [27], and ease of delivery by community-based facilitators with limited experience of the promoted skills [28].

The non-profit Digital Green Foundation pioneered the use of low-cost participatory videos and facilitated discussions with women’s self-help groups to strengthen agricultural extension. The organisation is now experimenting with leveraging its platform for MIYCN-related BCC in Odisha, India, with two partners: the Voluntary Association for Rural Reconstruction and Appropriate Technology (VARRAT), a local organisation working with self-help groups and responsible for project implementation; and the USAID-funded Strengthening Partnerships, Results and Innovations in Nutrition Globally (SPRING) project, responsible for the provision of technical assistance on MIYCN-related BCC. We conducted a feasibility study of the pilot project to shed light on key considerations for leveraging an agriculture platform for MIYCN.

This paper reports on this feasibility study and responds to the notable gap in the literature on the implementation of agriculture-nutrition strategies to address undernutrition [29,30]. It has three objectives:

1. to examine the process of integrating MIYCN into the existing low-cost, participatory, video-based agricultural extension platform targeted to women’s self-help groups and compare the development and delivery of agriculture and nutrition content;
2. to assess the viability of promoting nutrition-specific actions through the platform, including acceptance and trial of promoted behaviours and diffusion of key messages; and
3. to assess synergies with government health and nutrition services.

## Materials and Methods

### The Intervention

#### The Digital Green approach to agricultural extension

**Fig 1:**
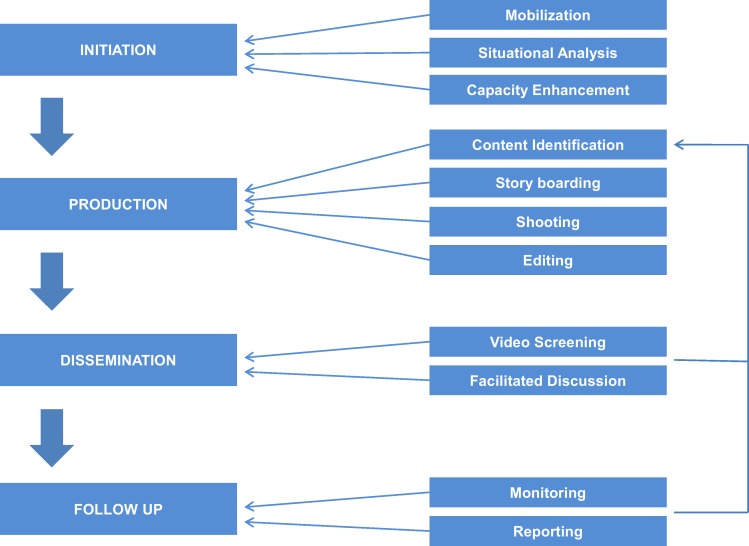
The Digital Green approach.

This approach builds on existing community organizations and public systems, and is aimed at amplifying their efforts at rural development and involves the following (Fig 1):

1. Mobilization of self-help groups and situational analysis to collectively identify community needs. Community members then are selected as programme personnel and trained on video production, dissemination, data management, and documentation.
2. Participatory identification of content and local production of low-cost videos to improve agricultural practices. Videos feature local community members speaking in local dialects narrating personal experiences and how adoption of best practice has benefited them. These protagonists are usually early adopters of innovations or best practices. Following production, each video’s content is reviewed by experts before it is approved for screening.
3. Dissemination through group discussions with self-help groups that use videos as a base for mediated instruction. At the screening, the mediator pauses the video at strategic points, encouraging the viewers to discuss, question, and reflect upon the video content.
4. Follow-up visits by the mediator to encourage and monitor adoption of disseminated practices.

In a pilot randomised controlled trial involving 16 villages, the Digital Green approach to agricultural extension increased adoption of specific agriculture practices seven-fold over the prevailing extension approach [21]. A World Bank collation of learnings from innovative usage of ICTs in rural development projects in South Asia affirms that by leveraging the Digital Green approach, community groups can “develop a localized, scalable model for agricultural extension, financial literacy, health and nutritional awareness and technology and livelihood training” [31]. Since 2008, Digital Green has reached over one million individuals across nine Indian States and in east and west Africa.

#### The Pilot Intervention to Explore Integration of MIYCN BCC into the Agricultural Extension Platform

Digital Green, in collaboration with SPRING and VARRAT, initiated a 10-month MIYCN BCC pilot intervention in 30 villages in Patna and Ghatgaon, two district blocks in the Keonjhar district of Odisha in October 2012. The pilot sought to (i) build the capacity of VARRAT in MIYCN and video production, dissemination, and facilitation skills; (ii) develop and disseminate 10 locally-produced videos that motivate adoption of recommended MIYCN behaviours; and (iii) assess the feasibility and acceptability of using the Digital Green platform and approach for promoting MIYCN-related behaviours among participants of women’s self-help groups. The pilot also aimed to develop synergies with existing government-funded frontline health services to ensure consistency of nutrition messages and create synergies with on-going efforts.

After extensive consultations with stakeholders and formative research, ten priority video themes were generated (Box 1) and a ‘package-of-practice’ for each theme was developed, detailing key behaviours and messages to be communicated. A nutrition expert based in Odisha provided technical support for storyboard and script development, video production, and mediated discussions, and ensured the overall technical accuracy of the messages. With the assistance of government frontline health workers, local people who were thought to practice some of the promoted MIYCN behaviours were recruited from villages in the intervention area to ‘star’ in videos. Frontline health workers typically featured as nutrition and health educators in these videos. The Digital Green approach to monitoring adoption of promoted practices also was modified to capture both experimentation with promoted MIYCN practices and the diffusion of messages promoted in the videos to non-viewers. The pilot videos were disseminated beginning in May 2013. In each of the 30 intervention villages, two self-help groups were included and shown two MIYCN-related videos per month (available at www.digitalgreen.org).

**Box 1. The ten MIYCN topics covered in the intervention.**

1. Importance of hand-washing with soap
2. Importance of the first 1000 days
3. Importance of iron-folic acid supplements during adolescence and pregnancy
4. Maternal diet and food taboos
5. Maternal workload during pregnancy and breastfeeding
6. Exclusive breastfeeding for the first six months
7. Accommodating breastfeeding for working mothers
8. Introduction of complementary feeding
9. Age appropriate complementary feeding for babies six to 24 months
10. Importance of strategies to improve dietary diversity

### Methods

#### Participants

The 30 intervention villages were purposefully selected to prioritise those with self-help groups active with the Digital Green programme and which included a larger proportion of pregnant and lactating women, and to represent variability in the population prevalence of Scheduled Castes and Scheduled Tribes (SC/ST) and remoteness. For the feasibility study, 15 of the 30 pilot intervention villages were included. These villages were chosen by further stratifying the 30 pilot villages by the predominance of SC/ST: high (SC/ST population greater than 90%); medium (SC/ST population between 50–75%) and low (SC/ST population below 40%). Compared to the rest of the Indian population, SC/ST suffer a higher burden of undernutrition [32] and have poorer access to and utilization of health services [32,33]. The sampling frame sought to capture this variation. From each stratum, five villages that participated in the pilot were randomly selected and, when selected villages had three or more women’s self-help groups, two groups were chosen at random (Table 1). The study was introduced to members of these self-help groups by VARRAT programme personnel.

**Table 1:** Sampling scheme and participants.

**30 intervention villages**		
Stratification of villages by prevalence of scheduled castes and tribes (SC/ST)		
HIGH SC/ST (≥ 90%)	MEDIUM SC/ST (50–70%)	LOW SC/ST (≤ 40%)
Random sampling of five villages	Random sampling of five villages	All five villages selected
Purposeful selection of 10–15 self-help group pilot intervention participants in each strata		
Household participants:	Household participants:	Household participants:
• 14 self-help group members ○ 3 pregnant women ○ 2 lactating women ○ 3 complementary feeding mothers ○ 2 mothers of adolescent girls ○ 4 other women • 10 Mothers-in-law • 14 Husbands	• 13 self-help group members ○ 2 pregnant women ○ 2 lactating women ○ 3 complementary feeding mothers ○ 2 mothers of adolescent girls ○ 4 other women • 12 Mothers-in-law • 12 Husbands	• 15 self-help group members ○ 3 pregnant women ○ 2 lactating women ○ 4 complementary feeding mothers ○ 2 mothers of adolescent girls ○ 4 other women • 13 Mothers-in-law • 12 Husbands
**Other study participants**		
• 16 VARRAT field-level programme personnel ○ 4 Community Resource Persons ○ 12 Community Service Providers • 7 Protagonists who featured in MIYCN videos • 6 Key stakeholders from partner organisations (SPRING, Digital Green, and VARRAT) • 7 Frontline health workers		

Abbreviations used: MIYCN: maternal, infant, and young child nutrition; SC/ST: Scheduled Castes and Scheduled Tribes; SPRING: USAID-funded Strengthening Partnerships, Results and Innovations in Nutrition Globally Project; VARRAT: Voluntary Association for Rural Reconstruction and Appropriate Technology.

Within each stratum, we recruited a purposive sample of about 14 women from women’s self-help groups, with an aim to include a range of pregnant women, lactating mothers, women with children between 6–24 months of age, mothers of adolescent girls, and women who do not fall into any of these categories. In those households where study participants had spouses and/or mothers-in-law, these members were included as well. The sample also incorporated data collection with persons involved in developing and implementing the intervention, including VARRAT programme personnel involved with relevant field operations (Community Resource Persons responsible for video production and Community Service Providers responsible for mediated discussion and follow-up behaviour monitoring visits), protagonists who acted in the videos, and other key stakeholders from VARRAT, SPRING, and Digital Green, as well as two frontline health workers from each stratum. All individuals invited to participate agreed to take part.

#### Data collection and analyses

A range of methods was used to collect data, including structured observations of disseminations, in-depth interviews, nutrition knowledge tests, and a social network questionnaire to assess the diffusion of MICYN messages (Table 2). Data collection tools were designed to capture the nuances of factors affecting intervention feasibility and are available as supporting information (S1–S9 Files). All tools were pilot tested by the local research team and subsequently revised to improve the clarity of questions. Data were collected face-to-face over a four week period in 2013 by two members of our research team with expertise in qualitative methods (AM and TR) and seven trained field researchers (six female, one male). All field researchers were fluent in Odiya and had university qualifications and at least five years of experience with qualitative data collection.

**Table 2:** Research methods.

Study participants	Data collection method / tool administered
Household participants	
Self-help group members	• In-depth interview covering acceptance, comprehension, and retention of MIYCN messages, as well as Community Service Providers’ capacity in mediation • Structured questionnaire on diffusion of MIYCN messages (including follow-up through to the third degree recipient) • Knowledge test related to the first seven MIYCN video topics^a^
Husbands and mothers-in-law	• Semi-structured interview covering knowledge and perception of the video disseminations and acceptance of trials of new behaviors
VARRAT project personnel	
Community Service Providers	• In-depth interviews covering capacity issues, experience in disseminating video messages, acceptance of content, process of adoption checks, and challenges in the model • Knowledge test related to the 10 MIYCN video topics • Structured observations of video disseminations
Community Resource Persons	• In-depth interviews covering capacity issues, experience in video production and challenges in adapting the MIYCN model in the existing agriculture extension platform • Knowledge test related to the 10 MIYCN video topics
Protagonists	• In-depth interviews covering recruitment, production process, diffusion of MIYCN messages, and perception of the model
Key stakeholders from SPRING, Digital Green, and VARRAT	• Key informant interviews covering roles, responsibilities and relationships among SPRING, Digital Green, and VARRAT; the relationship of VARRAT with intervention communities; Community Resource Person and Community Service Provider capacity issues; content adaptation; operational challenges; monitoring and assessment; strategic issues
Frontline health workers	• In-depth interviews covering perceptions about the model and capabilities of Community Service Providers and Community Resource Persons, acceptance of video messages, relationship with VARRAT and Digital Green, and change in community dynamics and nutrition behaviour

^a^ Nutrition knowledge tests administered to self-help group members related only to the first seven topics as the dissemination of the final three videos were not complete in some sample villages. Community Resource Persons and Community Service Providers were asked questions about all MIYCN video topics, as they were trained in all 10.

Abbreviations used: MIYCN: maternal, infant, and young child nutrition; SPRING: USAID-funded Strengthening Partnerships, Results and Innovations in Nutrition Globally Project; VARRAT: Voluntary Association for Rural Reconstruction and Appropriate Technology.

Data were collected privately from household participants and protagonists in their homes, and from other participants at their place of work. Interviews lasted about two hours and were audio-recorded and later transcribed. Interviews with key stakeholders from partner organizations were conducted in English. All other interviews were conducted and transcribed in Odiya and then translated to English. Data saturation was considered when determining sample sizes and reviewed iteratively as data collection and transcription proceeded. Nutrition knowledge tests and the social network questionnaire were administered at the time of the interview. No repeat interviews were conducted.

Interview data were coded using the NVivo software programme (QSR International Pty Ltd. Version 10, 2012). Seven trained study staff coded the data following an *a priori* descriptive code list derived from the interview guides. NVivo’s matrix function was used to analyse coded text. Quotes presented in this paper are illustrative of general trends in the data. Data from the diffusion questionnaire were analysed in Python-networkX [34] and Microsoft Excel (2013) and visualised using Gephi [35]. Data from one high stratum household (self-help group member, husband, and mother-in-law) were excluded from qualitative analysis and diffusion analyses due to poor data quality. Data collected from observations and nutrition knowledge tests were saved in a separate Microsoft Excel file and reviewed descriptively.

#### Ethics Statement

The institutional review boards of International Food Policy Research Institute (Washington, DC, USA) and the Indian Institute of Public Health (Bhubaneswar, India) reviewed and approved this study (#IIPHB-IEC: -2013/021). Participants provided their informed, verbal consent prior to participation. Verbal consent was deemed appropriate given the low literacy rates in Odisha, particularly among women in tribal areas. The consent process was certified and documented by the researcher obtaining consent with a signed and dated statement. This procedure was specifically approved by both institutional review boards.

## Results

The existing low-cost, participatory, video-enabled agricultural extension programme has many strengths as a platform for disseminating MIYCN BCC. Importantly, the nutrition intervention responded to the high demand for ‘health education’ in communities. Our respondents mentioned that the communities felt “*health was at their hands’ distance*, *but they were ignorant*” about it. According to all self-help group members interviewed, the intervention was the main, if not the only, source of information related to health and nutrition targeted broadly at the community level.

*“We didn’t know about it [health and nutrition] from anywhere else*. *After watching the video we got to know*.*”* (Self-help group member (pregnant woman), Ghatgaon)

The intervention was met with high levels of enthusiasm and acceptability from self-help group members, husbands, and mothers-in-law, as well as government frontline health workers. In principle, mothers-in-law and husbands of self-help group members do not object to women attending the dissemination sessions. In fact, some mothers-in-law started accompanying their daughters-in-law to the nutrition sessions. The diverse range of respondents unequivocally regarded Community Resource Persons (who produce videos) and Community Service Providers (who disseminate videos and conduct follow-up visits) as credible sources of knowledge related to health and nutrition (regardless of the agent’s sex). Whether this is because of their familiarity with existing agricultural programming or the nutrition content of the videos is difficult to determine.

The results shed light on issues and opportunities related to leveraging an existing participatory, video-based agriculture extension programme for MIYCN-related BCC. In the sections below, we present key results by research objective. Although the household/self-help group member interviews were stratified both by proportion of SC/ST households in the community, and by the ‘type’ of self-help group member in the household, there were no appreciable differences in the nature of responses along either stratification axis. For this reason, we present aggregated findings.

### Process of Integrating MIYCN into the Agricultural Extension Platform

#### Identification of MIYCN Content

Despite a high demand for health information, interviews with programme personnel revealed content identification to be more challenging for nutrition as compared to agriculture. Unlike agriculture, a ‘tangible topic’–where people watch crops grow or fail and experience profit or loss–nutrition is more abstract, with longer and more complex cause-and-effect linkages. As such, community members were less able to explain the specific health and nutrition practices with which they would like support. Programme personnel stressed the benefits of mediated discussions during dissemination sessions to help identify demand for new content, specific information needs, and community members with positive deviant behaviours for health and nutrition (Table 3).

**Table 3:** Process of integrating MIYCN into the agricultural extension platform.

Themes	Quotes
Identification of MIYCN content	• *“If there is some problem in terms of insect attack [on crops]*, *then they [farmers] will let you know*, *or if they wanted to do something new or maybe something existing in a different way*, *then they will ask you… But in nutrition videos*, *the problem at this point of time is that the people may not articulate [what issues they need information on]*.*”* (Key Stakeholder, Digital Green) • *“As they screened these ten [videos]*, *people [in the disseminations have] just spoke up and talked about their own personal experience*. *And I know that [Digital Green] and VARRAT [staff] have been taking notes and the Community Service Providers who know their communities well have been taking notes about people in the community that are practicing [the promoted behaviours]… I think the subsequent videos might look a lot… different*, *feel different*.” (Key Stakeholder, SPRING)
Production of MIYCN-content videos	• *“In agriculture we were able to [produce videos] easily*. *But in this [MIYCN intervention] some more materials were required*. *More people were there and more shots were required to take*. *In that [agriculture]*, *you show how he is doing cultivation*, *how he is ploughing*, *etc*. *But in this [nutrition] you show about food [and] different types of items*. *These are the differences*.” (Community Resource Person, VARRAT) • *“[The] problem [is that] while editing we saw that there were so many clips*. *First*, *we felt nervous by watching that only–so many clips how would we edit*! *Then after watching two to three times*, *[we] did not feel like that [any]more*.” (Community Resource Person, VARRAT)
	• *“Most of them are farmers in our area*. *[For the agriculture videos] it was not so difficult to find a farmer*. *It is done easily*. *But in [the] case of nutrition [video production]*, *it is [a] little difficult to find a mother having [a] six months old baby for the videos of supplementary food of a six months old baby*.” (Community Resource Person, VARRAT)
Dissemination and mediation of the MIYCN-content videos	• *“They [self-help group members] told [me] that they had forgotten [key messages on iron and folic acid supplementation and diet during pregnancy]*. *Suppose there are six or seven points and they only remember two or three… ‘This [topic] is quite interesting’ and ‘repeat again’ they say*.” (Community Service Provider, VARRAT) • *“We have to explain more in videos on nutrition*, *and it is comparatively lesser in agricultural videos*.” (Community Service Provider, VARRAT)
Follow-up visits to monitor uptake of the disseminated MIYCN practices	• *“In farming*, *they have already done it [the promoted behaviour] and we go to the field to see whether this much distance [between rows of seeds] is maintained or not*. *In nutrition*, *if someone lies too you*, *[you] can’t know immediately*. *Those things we have to understand and see*.” (Community Service Provider, VARRAT)

Abbreviations used: MIYCN: maternal, infant, and young child nutrition; SPRING: USAID-funded Strengthening Partnerships, Results and Innovations in Nutrition Globally Project; VARRAT: Voluntary Association for Rural Reconstruction and Appropriate Technology.

#### Production of MIYCN-content Videos

Personnel involved with video production, primarily the Community Resource Persons and the nutrition technical experts, described the process as more complex and time-intensive for nutrition than agriculture (Table 3). Storyboards had to be carefully designed to ensure communication of each topic’s multidimensional package-of-practice. While economic determinants are predominant in agriculture, for MIYCN, sociocultural determinants also play a critical role because the body, central to MIYCN BCC themes, is a site for intense cultural elaboration in a way that agriculture is not. This made creating storyboards for effective ‘messaging’ more challenging, as videos needed to address not only economic considerations but also embed nutrition and health messages in sociocultural context. Thus, nutrition videos tended to have a larger cast of characters and several layers of messages requiring greater ingenuity to develop.

The complexity of the videos, in turn, increased the complexity of site selection and casting (Table 3). Whereas agriculture videos typically featured one or two people demonstrating or describing a discrete practice in a field, storylines for the MIYCN videos often involved multiple people interacting inside the home, requiring additional technical consideration for issues around voice modulation, lighting and editing. Identification and selection of actors was challenging given that the protagonist had to be in the particular life stage featured in the video (e.g. pregnant, lactating) and all actors had to be confident and articulate speakers, which was difficult to find amongst young women in remote tribal areas. Government frontline health workers often supported this process by recommending women that had adopted positive MIYCN practices from the villages they serve.

#### Dissemination and Mediation of the MIYCN-content Videos

Self-help group members had strong belief in the information communicated and found the MIYCN videos to be compelling and appropriately paced. Despite the positive response, Community Service Providers responsible for screening the videos and mediating discussions felt that self-help group members required greater explanation of nutrition compared to agriculture information. Furthermore, some self-help group members and Community Service Providers felt that MIYCN videos should be screened multiple times to help clarify and solidify messages (Table 3).

VARRAT field-level programme personnel related that they were excited to learn about health and nutrition and to share this new information in local communities. However, nutrition knowledge tests revealed that their knowledge on MIYCN required further strengthening, perhaps related to the nascent nature of the MIYCN component compared to agriculture. Whilst all field staff had an accurate understanding of optimal breastfeeding practices and all but one were able to correctly explain the first 1000 days, fewer were knowledgeable of appropriate complimentary feeding, care during pregnancy, use of iron and folic acid supplementation, and hand-washing. Descriptive data from structured observations (available on request) revealed facilitation skills varied among Community Service Providers, with about half struggling to adequately answer self-help group members’ health and nutrition questions.

#### Follow-up Visits to Monitor Experimentation with and Diffusion of the Promoted MIYCN Practices

As previously noted, the video-based participatory agriculture extension programme includes several layers of checks to assess if a particular promoted agricultural practice has been adopted. For the nutrition pilot, this verification process was adapted to assess both experimentation with promoted MIYCN practices and ‘diffusion’ of messages, i.e. sharing the information disseminated in the video sessions with others. Follow-up visits to households to reinforce messages and maintain community engagement are some of the advantages of the Digital Green approach to agricultural extension. However, unlike agriculture, where follow-up visits normally involve examining a farmer’s fields for visible signs of adoption, trialling of MIYCN behaviours is largely unobservable and checks for behaviour change depend on self-reporting. Reliance on probing and triangulation using proxies and other techniques by the Community Service Providers, whose nutrition-relevant knowledge is fairly rudimentary, can be problematic (Table 3).

### Viability of Promoting MIYCN Practices through the Agricultural Extension Platform

#### Acceptance of MIYCN Messages

The integration of nutrition content into the video disseminations received high praise from self-help group members and their families (Table 4). While acknowledging the sensitive nature of many nutrition and health topics, the majority of respondents felt that videos were interesting and covered acceptable topics. Self-help group participants reported ‘liking’ MIYCN topics and that learning about nutrition ‘feels good’.

**Table 4:** Viability of promoting nutrition-specific actions through the agricultural extension platform.

Themes	Quotes
Acceptance of MIYCN messages	*• “It (the video programme) is very nice*. *The daughters-in-law are learning by it*. *They tell us how to take care of babies*, *so it is nice*. *Our daughter-in-law goes to watch [the] video on the day when it is played*.” (Mother-in-law of a self-help group member, Patna) • *“Earlier*, *it used to be embarrassing*. *When somebody used to tell anything*, *it was embarrassing*. *But now we don’t feel embarrassed*.” (Self-help group member (other category), Patna) • *“In the video ‘Workload during pregnancy’*, *[a] mother-in-law said that they (the older generation) have given birth to many children [while] taking the burden of work*. *Why should their daughters-in-law only rest [during pregnancy]? Then she said*, *‘Here is the [frontline health worker]*. *Let’s ask her.’”* (Community Service Provider, VARRAT)
Comprehension and retention of MIYCN messages	*• “They are showing everything in the video*. *[The] doctor only explains things in words and we listen to him*. *The difference [with the videos] is what we hear and what we see*.” (Self-help group member (lactating mother), Ghatgaon) • *“In detail*, *they show [the promoted practices] in [the] video*. *There is no difficulty in understanding… In [the] video*, *villagers act [and frontline health workers] explain [what to do]*, *and talk taking rest*, *while in TV they talk in a flow which is not understandable*.” (Self-help group member (other category), Patna) • *“He (Community Service Provider) speaks as a mother can understand.”* (Self-help group member (complementary feeding mother), Ghatgaon)
Experimentation with promoted MIYCN practices	*• “By seeing [the] videos*, *they are now neat and clean*. *Their diet has improved*. *Before there was [dietary] restriction*, *[but] now they do not deny [food]*. *They are having enough rice… I roam in the village*. *Earlier the village was so untidy*, *dirty and also*, *the clothes they were wearing were dirty*. *Now it’s clean*. *Even in the group*, *everybody sits wearing clean sarees*.” (Frontline health worker, Ghatgaon) • “*It is not possible for all to eat proper diet every day*. *It’s difficult to arrange all types of food for them*. *I mean*, *the poor cannot eat everything always.”* (Self-help group member (other category), Patna)

Abbreviations used: MIYCN: maternal, infant, and young child nutrition; VARRAT: Voluntary Association for Rural Reconstruction and Appropriate Technology.

A small subset of respondents expressed concerns about the acceptability of the approach in sharing certain health and nutrition information. In particular, the presence of men–including Community Service Providers–in the room during disseminations focused on pregnancy and breastfeeding made a small proportion of women feel ‘shy’, ‘embarrassed’, or ‘shame’. However, other women described overcoming initial discomfort during the course of the pilot and embracing the information shared (Table 4).

Some messages were found to conflict with traditional beliefs and norms. An example was the customary belief that if women take iron and folic acid supplements, childbirth will be more painful because the baby would grow larger. Another example is related to pregnant women’s consumption of ripe fruits which is believed to cause wounds in a baby’s head: “*By taking ripe fruits they think there would be ripe in the baby’s head”* (Self-help group member (lactating mother), Ghatgaon). As a result, inter-generational resistance to the messages disseminated was not uncommon. For example, while the recommendation for women to do less work while pregnant was well-received by pregnant women, several mothers-in-law pointed out that “*it wasn’t [like] this…in our times”* and that pregnant women should, as tradition dictated, shoulder a full workload.

According to both Community Service Providers and government frontline health workers, introduction of MIYCN information generated a communication momentum, with discussions on new and controversial topics occurring both during and after dissemination sessions. For example, Community Service Providers noted that sometimes mothers-in-law disagreed with the MIYCN messages communicated through the intervention and asked frontline health workers to confirm their accuracy (Table 4).

#### Comprehension and Retention of MIYCN Messages

Data from nutrition knowledge tests illustrated that self-help group members were familiar with some but not all of the major ideas promoted through the intervention (Table 5). Knowledge was highest on the topics of women’s need to eat more than usual during pregnancy and lactation, and the importance of exclusive breastfeeding. Whilst the majority of self-help group members correctly demonstrated hand washing, only a third could recall all three critical times for hand washing (i.e. before preparing food, eating, or handling a baby). Self-help group participants had less accurate knowledge of care during pregnancy and lactation, iron and folic acid supplementation, strategies to support exclusive breastfeeding by working mothers, and the importance of the first 1000 days. It is important to note that this study is not an impact evaluation and therefore, it is not possible to assess change in knowledge or to attribute such changes to the intervention.

**Table 5:** Number and percent of self-help group women demonstrating accurate knowledge of critical practices for MIYCN covered in the first seven dissemination sessions (*n* = 42).

Topic	(#)	(%)
Importance of hand-washing with soap		
Mentions washing before preparing/handling food	25	59.5
Mentions washing before eating	40	95.2
Mentions washing before handling baby	21	50.0
Mentions all three adoption points	14	33.3
Demonstrates hand-washing with clean, running water and soap	34	81.0
Importance of the first 1000 days		
Correct explanation of first 1000 days	13	31.0
Iron and folic acid supplementation		
Adolescent girls between 10–19 years of age should take IFA tablets	11	26.2
Pregnant women should take IFA tablets daily starting in the second trimester	20	47.6
Maternal diet and food taboos		
Pregnant woman should eat more than usual	36	85.7
Breastfeeding women should eat more than usual	36	85.7
Maternal workload during pregnancy and breastfeeding		
Need for rest during pregnancy	24	57.1
Exclusive breastfeeding for the first six months		
Timely initiation of breastfeeding within one hour of birth	31	73.8
Colostrum should be fed	37	88.1
Exclusive breastfeeding in the first six months	35	83.3
Baby should be fed on demand both day and night	37	88.1
Accommodating breastfeeding for working mothers		
Mentions two or more ways families can support breastfeeding by working mothers	20	47.6

Abbreviations used: IFA: iron and folic acid; MIYCN: maternal, infant, and young child nutrition.

Overall, videos were described by household participants, VARRAT field-level personnel, and frontline health workers as a powerful medium for sharing complex MIYCN messages (Table 4). Many of these respondents suggested that health and nutrition information presented through videos is easier for people to understand and remember than the same information communicated verbally. Further, several self-help group participants expressed that seeing local people in the videos made behaviour change seem more feasible and discussion led by the Community Service Providers helped clarify messages.

#### Experimentation with Promoted MIYCN Practices

Diverse sources, including household participants and programme personnel, described trials of recommended practices in intervention communities (Table 4). For each promoted MIYCN behaviour, at least 60% of respondents who had received information on it reported trying it. The behaviours most frequently tested were hand-washing and iron and folic acid supplementation; all respondents who received information about the benefits of hand-washing reported testing one or more action related to hand-washing (e.g. use of soap, rubbing palms together) and 86% those whom were eligible for iron and folic acid supplementation and received information about its importance reported taking tablets. Lending credibility to these data, government frontline health workers reported an upsurge in demand for iron and folic acid tablets and described witnessing positive examples of behaviour change in their communities, such as more hand-washing, better breastfeeding habits, and improved diets for pregnant women.

As with agriculture, economic factors contributed to the likelihood of sustained adoption of recommended MIYCN behaviours. In addition to financial costs of advised supplies such as soap and healthy foods, self-help group participants described non-financial costs, such as time and effort required to fetch water, particularly in the dry season (Table 4).

#### Diffusion of MIYCN Messages

We explored two dimensions of message diffusion through a structured questionnaire. First, we examined the social networks in which messages were spread through villages and the number of messages passed from one person to the next. We assessed if a self-help group member directly shared information with another person (first degree diffusion) and if the recipient of the message from the self-help group member (i.e. first degree recipient) shared the message with another person, which we refer to as second-degree diffusion. Information was elicited on seven videos that were disseminated throughout the study villages when the study was conducted.

**Fig 2:**
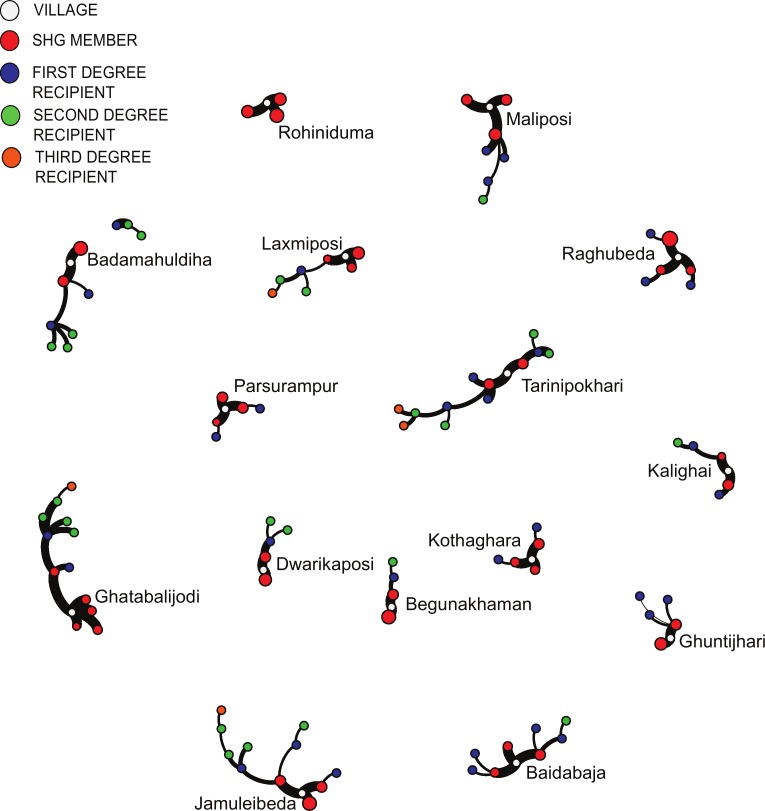
The weighted network of message diffusion, with link thickness proportional to the number of messages transmitted. Self-help group members that did not transmit any messages are not presented.

In all but one village, MIYCN messages were shared following dissemination sessions (Fig 2). About half of self-help group respondents (*n* = 22) confirmed they had shared information from any of the MIYCN videos they viewed with at least one person. Pathways of diffusion of MIYCN messages within communities were diverse. Exchanges of information between spouses and from daughter-in-law to mother-in-law were common. About a third of the sharing resulted in second-degree diffusion. However, two thirds of links were weak, with information from only one or two videos passed to either the first or second degree.

**Fig 3:**
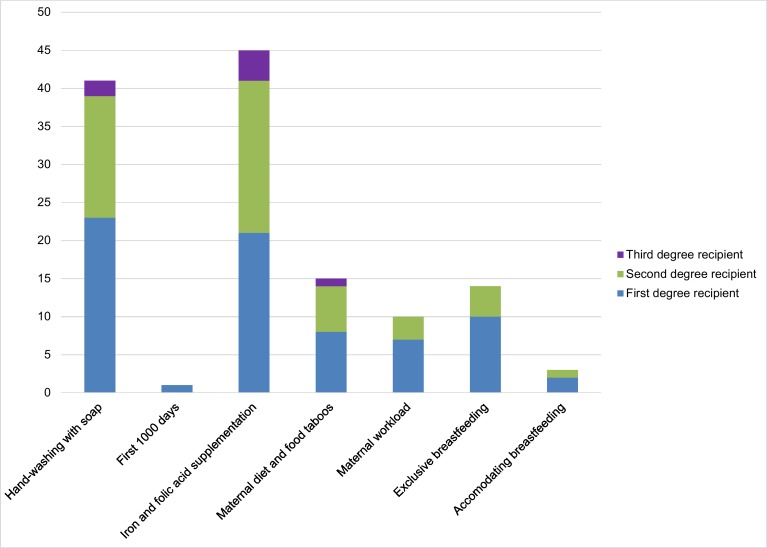
The extent of diffusion by video topic and role in the communication flow. Self-help group members that did not transmit any messages are not presented.

Second, we considered the extent of diffusion by MIYCN topic. Information related to the importance of iron and folic acid supplementation and the benefits of hand washing with soap–behaviours that require simple and discrete steps–emerged as the messages most commonly shared by self-help group members (Fig 3). These were followed by the messages on maternal diet and food taboos, the importance of exclusive breastfeeding, maternal workload during pregnancy, and accommodating breastfeeding for working mothers–actions that require a constellation of inter-related behaviour changes and may be dependent on others’ actions. Only one self-help group member passed on information about the first 1000 days.

### Synergies with Existing Frontline Health Services

#### Engagement with Frontline Health Workers

The pilot sought to proactively engage frontline health workers in development and delivery of the intervention in order to harmonize efforts with the existing health system–a prerequisite for sustainability and scaling-up. This strategy appears to have an important influence on the acceptability and credibility of the intervention. The frontline health workers interviewed unanimously expressed support for MIYCN videos. They valued being included in the training provided to VARRAT field-level programme personnel. They felt that the videos helped refresh their nutrition knowledge and considered programme personnel as a valuable resource (Table 6). In some instances, frontline workers mentioned that their supervisors suggested they watch the videos to understand specific MIYCN issues. Given the poor state of refresher training of frontline workers in India, the pilot nutrition intervention appears to partly fill this void.

**Table 6:** Synergies with existing frontline health services.

Themes	Quotes
Engagement with frontline health workers	*• “ From training we listen and come*. *Many things we used to forget*. *To see it again gives pleasure*. *[A] little more things stay in memory*. *We recollect it*. *Everything you cannot do always*. *[If] you have forgotten something*, *by seeing [the video] automatically it will come to your mind.”* (Frontline health worker, Ghatgaon)
Complementary approaches for delivering MIYCN BCC	*• “To explain [to] them (women)*, *it was very difficult*. *But by watching the video*, *it has become easy*.” (Frontline health worker, Ghatgaon) • *“What I am teaching them they are watching that on screen [during the video dissemination]*. *Hearing the same thing repeatedly*, *how can they go away from this?*” (Frontline health worker, Ghatgaon) • *“Earlier*, *we did not know about all these [things]*. *We used to take food without washing [our] hands*. *It (behaviour change) started*, *when my wife told [us] about [how] washing [our] hands with soap is good and that [it] prevents [us] from diseases… The nurse sisters have also told [us this same information]*. *We do also see people and learn*. *Thus it is being practiced*.” (Husband of a self-help group member, Patna) • *“There is difference*. *In Anganwadi [centres] (government centres for MIYCN and early childhood education)*, *they explain [MIYCN information] only to a mother; sometimes the mother-in-law accompanies her*, *but others are left [unaccompanied]*. *But by the Digital Green method*, *[the] mother*, *mother-in-law*, *daughter-in-law*, *father-in-law*, *husband*, *everybody can see [the video] at a time.”* (Community Service Provider, VARRAT)
Demand expansion for government health and nutrition services	*• “ One [of the] most important things I can say [is] that some of the aspects [of government health services] which have come up in the videos*, *were not practiced earlier [but are now being practiced]*. *Say for example [a frontline health] worker has to give an iron and folic acid tablet to all the adolescent girls once a week*, *which was not practiced all*. *Even the adolescents were not aware [that] they (frontline health workers) are supposed to give [out] the iron and folic acid tablets*. *So at least through this [programme]… the [frontline health] worker now feels her duty… “If I don’t give them [the iron and folic acid tablet]*, *they may ask me*, *‘why you are not giving?’” So that is now starting*, *so that is most important*. *The facilities [and] the benefits which were at the government level and were not reaching to the point*, *have now started reaching.”* (Key Stakeholder, VARRAT)

Abbreviations used: MIYCN: maternal, infant, and young child nutrition; VARRAT: Voluntary Association for Rural Reconstruction and Appropriate Technology.

#### Complementary Approaches for Delivering MIYCN BCC

Several frontline health workers said that they viewed the nutrition videos as job aids, reinforcing their efforts at promoting MIYCN within their communities. Household participants also emphasised that repeated exposure to the messages through different information channels supported comprehension and behaviour change (Table 6).

VARRAT field-level programme personnel mentioned several requests from frontline health workers for nutrition videos to be disseminated during ‘Village Health and Nutrition Days’, the health and nutrition fairs organized by the public health system once a month.

Frontline health workers expressed strong optimism about MIYCN BCC delivered through the Digital Green platform promoting improved MIYCN practices. Some felt a unique strength of the programme was that by targeting women of all ages, rather than solely pregnant women and mothers of young children, it allowed influential community and family members who would not traditionally engage with the frontline services to gain exposure to MIYCN messages (Table 6).

#### Demand Expansion for Government Health and Nutrition Services

A diverse set of study participants, including frontline health workers, reported increased awareness and uptake of government health and nutrition programmes following commencement of the pilot (Table 6). However, supply side challenges that could compromise the effectiveness of the intervention were identified, particularly in the availability and quality of government-provided food and micronutrient supplements. For example, one Community Service Provider said that after viewing the video on the importance of iron and folic acid supplementation, self-help group members reported that they were unable to access iron and folic acid tablets from frontline health workers. Another Community Service Provider described self-help group members complaining that *chhatua*–the supplementary food provided by the government–was inedible and worm-infested.

## Discussion

This paper examines the feasibility of delivering a MIYCN intervention through an existing participatory low-cost video platform for agricultural extension. The aim of the study was to bridge a key gap in the literature on *how* to leverage an agriculture platform to accelerate progress in reducing undernutrition through promotion of key MIYCN behaviours. Despite growing interest in this topic, most research on promoting nutrition through agriculture programming has focused on targeted interventions through the food supply, such as homestead food production and biofortification. To our knowledge, this study is the first to examine how a participatory, video-based agricultural extension programme could be leveraged as a delivery platform for nutrition BCC.

Our study showed it to be both possible and advantageous to promote MIYCN practices through participatory video-based agriculture platforms. The intervention benefited from high demand, trust, and acceptability by a wide range community stakeholders already familiar with the participatory agricultural extension programme. The positive reputation of the existing programme lent credibility to the nutrition intervention, even though some promoted MIYCN behaviours conflicted with existing practice. In the rural villages where the pilot was administered, the intervention was a principal source of health and nutrition information at the community level. From the perspective of participants, key strengths included: interest in MIYCN topics, appropriate pace and flow to dissemination sessions, sociocultural familiarity with the video cast, and opportunities for additional instruction and learning through facilitated discussion.

One of the most important findings for future multisectoral programmes for nutrition is the benefit of cultivating synergy between the intervention and local health systems responsible for delivering nutrition-specific programming. Frontline health workers were eager to collaborate on this project and their engagement broadened coverage by providing another trusted channel for spreading key MIYCN messages in communities. Further, they viewed the intervention as reinforcing their efforts and welcomed MIYCN videos as job aids. While community health workers are critical to health promotion and care in many low and middle income countries, previous research has shown frontline health services to face a number of challenges [36,37]. For example, in Odisha, where the intervention was implemented, many health workers do not meet minimum education requirements or receive required trainings [38]. Trainings, when offered, are often of poor quality. Further, many health workers overlook some intended responsibilities, particularly those related to education and counselling, and focus on activities that directly lead to compensation and product distribution [38]. The results of this pilot suggest substantial potential for interventions of this nature to boost demand for government nutrition- and health-related services, which may increase both frontline health workers’ capacity as well as accountability to fulfil the full range of work responsibilities. As has been observed previously in evaluations of interventions to improve uptake of health services in developing countries, this study found that it is not sufficient to assume that existing services are adequate and ready to address a potential increase in demand induced by positive behaviour change [39–41]. Rather, programme designers should explicitly consider how to create synergies with local health systems in order to strengthen the delivery of related services (e.g. training health workers in MIYCN) and resolve supply-side issues (e.g. reducing possible bottlenecks and building constructive mechanisms for grievance and redress).

Our study identified several aspects of participatory agricultural extension delivered through women’s self-help groups that require special consideration with respect to integrating nutrition BCC and maximising impact. First, assessing demand and crafting MIYCN BCC responsive to the local context was found to be more complex for nutrition compared to agriculture. Community members are less able to articulate specific nutrition topics and practices with which they would like support, and selection of positive deviant protagonists to feature in the videos is less straightforward given the complex, abstract, and unobservable nature of many nutrition and health behaviours. However, questions and stories shared by self-help group members as the pilot progressed suggest the potential for more iterative, community-driven content generation as learning about and discussing sensitive health concepts becomes more common. Further, increased trialling and adoption of the promoted MIYCN practices may facilitate identification and recruitment of protagonists for videos.

Second, we found monitoring the adoption of promoted behaviours to be more complex for nutrition than agriculture. Reliance on self-reporting and indirect questioning likely compromises accuracy [42] and the development of appropriate probes requires solid nutrition knowledge. Valid tools to monitor behaviour change by field-based programme personnel and on-going capacity development for implementing partners are needed to increase the validity of monitoring efforts for nutrition programmes.

Third, because only a minority of self-help group members represent the target population of pregnant and lactating women, the ultimate impact of the intervention will depend on the effectiveness of group members to diffuse key messages to stimulate social change. Although operating through self-help groups is an indirect approach to reaching young mothers, it has the benefit of engaging other important household decision-makers–such as current and future mothers-in-law–who often have considerable control over women’s labour and childcare practices [38]. These stakeholders may also have higher status in the village and thus greater social influence. Limited sharing of messages during the pilot suggests a need to fortify diffusion mechanisms. Efforts are needed to support self-help groups to become MIYCN change agents to help young mothers to negotiate sociocultural barriers, rethink cultural norms with detrimental impacts on child health and nutrition, adopt optimal MIYCN practices, ensure delivery of government health services, and bring MIYCN onto the community agenda. Participatory, dialogue-based interventions with women's groups work by providing a forum for communities to develop a common understanding of problems, as well as locally acceptable and sustainable strategies to address them. Such interventions have been shown to improve essential new-born care practices, hygienic deliveries, prevalence of neonatal mortality, and postnatal depression [43]; uptake of social protection programs [44]; women’s assets [45]; and women’s ability to link to markets and inputs [46]. Further assessments of conditions necessary for self-help groups to become MIYCN champions are necessary.

There are some limitations to this work. During this pilot phase a single video per topic was produced and each self-help group viewed the video only once. Intensity and duration of exposure to interventions is critical to bring meaningful behaviour change [47] and Digital Green typically develops and disseminates several videos per topic to support learning [21]. The study was designed to test the feasibility of the intervention and therefore no claims of attribution can be made. However, information gathered in this study on intervention development will help inform the design of more contextually appropriate and responsive nutrition-sensitive activities and programmes. We plan to test the effectiveness and cost-effectiveness of the strengthened intervention, informed by this feasibility study, in the next phase. Further, the pilot was conducted in one location in India, and findings may not be generalisable in other localities; however, with appropriate caution, these lessons can support other efforts seeking to deliver MIYCN BCC through an existing agricultural programme.

Despite many unknowns about how to integrate nutrition into programmes in related sectors, major investments in multisectoral action are already being made [3,4]. Calls to action so far have focused on the need for theory-based impact evaluations of nutrition-sensitive programmes [11,30]. However, following the approach of this study, future work also should include documentation and dissemination of programme experiences in order to inform new initiatives.

## Conclusion

A stronger understanding of how agricultural platforms can be leveraged for nutrition is needed to improve the coverage and scale of nutrition interventions targeting at-risk populations. Our study has illustrated some opportunities and complexities in deploying an innovative agriculture extension platform to deliver MIYCN BCC. Compared to agriculture, we found nutrition content to require more time and creativity to develop and technical support to deliver. However, the intervention, which used a participatory, locally-appropriate, video-based approach and targeted women’s groups, was well received by rural communities and viewed as complementary to existing frontline health services. Key lessons learned include the benefits of and need for collaboration with existing health services to couple programming with ongoing nutrition-specific activities; continued technical support for implementing partners to ensure accuracy of nutrition content; engagement with local cultural norms and beliefs to support positive MIYCN-related behaviour change; empowerment of women’s group members to strengthen their participation during and beyond the intervention lifecycle; and enhancement of message diffusion mechanisms to reach pregnant women and mothers of young children at scale. Our experience developing and delivering this agriculture-nutrition pilot intervention is one of the first to be documented and provides timely information upon which new multisectoral efforts can benefit and build.

## Supporting Information

S1 File Guide for semi-structured interviews with household participants. (PDF)

S2 File Guide for key informant interviews with Digital Green staff. (PDF)

S3 File Guide for in-depth interviews with VARRAT Community Resource Persons. (PDF)

S4 File Guide for in-depth interviews with VARRAT Community Service Providers. (PDF)

S5 File Guide for in-depth interviews with frontline health workers. (PDF)

S6 File Guide for in-depth interviews with video protagonists. (PDF)

S7 File Nutrition knowledge test related to the maternal, infant, and young child nutrition video topics. (PDF)

S8 File Structured questionnaire on diffusion of maternal, infant, and young child nutrition messages. (PDF)

S9 File Guide for structured observations of video disseminations in villages. (PDF)
