# Supplementary material for: Adapting Agriculture Platforms for Nutrition: A Case Study of a Participatory, Video-Based Agricultural Extension Platform in India
Source: PLoS One. 2016 Oct 13;11(10):e0164002. doi: 10.1371/journal.pone.0164002 (PMC5063370; doi:10.1371/journal.pone.0164002)
Supplement: S1 File — (PDF) [file pone.0164002.s001.pdf]

HOUSEHOLD INTERVIEW MODULES

Instructions: The researcher should elicit responses around the content areas outlined below. The researcher should look to the main points to guide conversation, in order to ensure that the relevant information is captured; however, it is important that the interviewer should avoid simply converting these subject headings into questions. The themes outlined below should be treated as 'conversation guides' that can facilitate a guided conversation between the researcher and the informant, focused on the main research themes. It is imperative that you obtain full, rich answers from the interviewee: always ask 'why?' and seek deeper explanations. Short, one-sentence answers are not sufficient. Finally, please remember that you must adjust the vocabulary according to the type of informant, while retaining the original meaning.

Modules included:

Exposure and retention matrix
Module 1: for SHG members
Module 2: for SHG household husbands
Module 3: for SHG household resident senior women (mothers-in-law)

Preliminary Information

Date of interview:

Village: Name of informant:

Pregnant: Yes / No /Don't know

Name of Husband:

Name of Mother-in-law:

**Number of children:**	**0**	**1**	**2**	**3**	**4**	**4+**

	**Age**		**Sex**	
**Child name**	**Years**	**Months**	**M**	**F**

EXPOSURE AND RETENTION MATRIX: PLEASE BEGIN THE INTERVIEW BY COMPLETING COLUMNS 3-5

No.	Theme	Number of times seen	Approx. how long before interview?	If not seen, reasons for not attending dissemination	Main points recalled (from question 4)
1	Importance of hand washing with soap				
2	Importance of the first 1000 days				
3	Importance of IFA supplementation during adolescence and pregnancy				
4	Maternal diet and food taboos				
5	Maternal workload during pregnancy and breastfeeding				

**6**	**Exclusive breastfeeding for the first 6 months**				
**7**	**Accommodating breastfeeding for working mothers**				
**8**	**Introduction of complementary feeding, CHATUA**				
**9**	**Complementary feeding (6 – 24 months): quantity, frequency, diversity, consistency**				
**10**	**Importance of and strategies to improve dietary diversity**				

HH MODULE 1: SHG MEMBERS

Assess the acceptance by SHG members of the DG approach for delivery of MIYCN messages as well as perceptions of the strengths and weaknesses of using that approach. Note: the interviewer should make sure that it is always clear which video she/he is asking about by specifying/describing the topic.

1) Ask the informant to tell you about the nutrition disseminations she has attended (you can use the completed table to prompt). Use the following probes to guide your conversation:

    - Please ask the informant to share their opinion of the nutrition dissemination(s) she has attended?
        - Suggested prompts if the informant is not responsive: protagonists, style of video, style of dissemination, believability, usefulness, feasibility (can she do what is asked?)
    - Please ask her to tell you:
        - Which one she liked best, and why (if she has attended more than one)
        - What was her favourite part and why?
        - What does she remember best about the dissemination? Why?
        - Was there any part in any video that she disliked or which made her feel uncomfortable or ashamed, and if so, why?

Explore informants' perspectives on CSP capacity to effectively deliver and disseminate nutrition knowledge through the DG platform

2) Ask the informant to discuss the CSPs who have carried out nutrition disseminations. Use the following probes to guide your conversation:

    - What is the informant's opinion of the CSP's nutrition knowledge?
        - Does the CSP seem to know the material well?
        - Are explanations provided by the CSP easy or hard to understand?
    - Do participants ask questions in the disseminations?
        - Is the CSP able to respond to questions?
        - Has the informant asked questions of the CSP? What was the response?

    - If unable to answer a question, what action does the CSP take?

- Are SHG members encouraged to participate actively in the disseminations?
    - If so, how are they encouraged and what form does this participation take?

- Do CSPs make time outside of the dissemination sessions for SHG members [to…discuss issues in the dissemination videos?]? Is there any time for consultation after the dissemination sessions or at other times?

- Ask the informant if are any particular traits of the CSP which either help or hinder the intervention or presentation of certain topics. Please prompt for gender if not mentioned.

- Ask the informant if there are any times when they would rather receive information from a woman (or man)

- Ask the informant if there is any other person she would rather receive this information from

- Ask the informant if she knows about VARRAT, and what they do in her community.
    - If she does, ask for her perspective on the overall relationship between VARRAT and the community.

Record SHG members' experiences with trials of new behaviors and identify their motivations for experimenting (or not) with new behaviors

3) Please refer to the disseminations attended, as recorded at the start of the interview. For each of the dissemination topics which the SHG member has been exposed to, you should ask the following sequence of questions:

- What was the practice/s which was promoted in the dissemination session?

- Has the interviewee tested any of the behaviors or practices in their household?

- If successful, did the interviewee continue to use the recommended behavior or practice? Why or why not?

- If the interviewee has knowledge of practices or recommended behaviors but has not tested them in their household, why not?

- Were there obstacles to testing those behaviors, or were they unwilling or uninterested to implement the practices in question?

- If she has adopted the practice, please explore:

- If she adapted the practice to her reality/local setting (i.e. substituting cheaper soap, etc.) to make it feasible
- What value does the interviewee place on the information shared? Do they find this information useful in their daily life?
- Whether she thinks the practice is effective and why;
- Whether she finds the practice easy, and why (please ensure that you cover the full gamut, from pragmatic issues through to socio-cultural logic);
- Whether she finds it difficult, and why (please ensure that you cover the full gamut, from pragmatic issues through to socio-cultural logic);
- Whether or not other household members are supportive of the adoption – and why or why not;
- How long she thinks she will be able to continue doing the practice.

Explore retention and comprehension of video content by SHG member attendees.

4) Ask the informant which disseminations she has seen, how many times she has seen them, and confirm that this information concurs with the information tabulated in the matrix at the start of the interview.
5) Ask the informant to tell you all the information she can recall from each dissemination she has attended. You can do this by asking her to describe each video, and to tell you what the video and dissemination session asked her to do. Please note the main messages in the matrix at the beginning of the interview guide.

Understand intra-household/ community diffusion effects, if any, of the intervention on the knowledge and attitudes of households that do not participate in video disseminations as well as on community health and nutrition workers. NB this research question will be systematically explored using the snowball and SNA approaches and tools. In the SHG interview, we are principally concerned with identifying potential first-level subjects for the SNA exercise.

6) Please ask the SHG member whether she has shared any information, knowledge or experience learned in the nutrition dissemination sessions with anyone, either within or outside her household.

- If not, ask her why she hasn't shared the information. Probes:
    - Lack of opportunity
    - Lack of interest
    - Other reasons (describe)

- If she has shared information, knowledge or experience with anyone in her social network (including family), please ask:
    - What she has shared;
    - With whom she has shared it;
    - Why she decided the share the information, knowledge or experience.
- Please ask the respondent whether she has ever had any conversations about MIYCN messaging, disseminations or nutrition videos with frontline community health or nutrition personnel (AWWs, ASHAs or ANM for example). If she has, please explore the following:
    - What topics were discussed;
    - Why these issues were discussed with the health or nutrition worker;
    - What were the health or nutrition worker's views on the messaging offered in the dissemination session(s)? Were there any messages or issues they disagreed with?
- Please ask the informant whether she has heard any of the nutrition messages in any context other than a dissemination session attended by her. If yes, please explore the following:
    - Which messages?
    - What was the source?
- Please explore the question of intra-group and intra-community social dynamics, with particular reference to:
    - SHG members: What are the relations like within the SHG? Do particular social relations in the SHG ever make dissemination sessions harder or less comfortable?

HH MODULE 2: SHG MEMBER HUSBANDS

1. Knowledge and opinion of program

    a. Please ask the husband to tell you what he knows about the VARRAT program, being careful to distinguish between the nutrition work and the previous agriculture intervention.

        - What is his opinion of the program? Why?
        - Does he know which videos his wife has watched? Has his wife shared any of the messages with him?
        - Has he shared any of the messages with anyone else?
        - What does he think of the dissemination approach?

2. Acceptance of spousal participation in the nutrition disseminations

    a. Please explore the issue of acceptability of his wife's participation

        - Does he have any problems with her attendance at disseminations or other activities?

3. Acceptance of new nutrition behaviors in household- you can refer to the above question about what nutrition messages the wife has shared with him and ask questions specifically about each practices.

    a. Find out his thoughts on the recommended practices.

        - Does he find them believable, feasible?
        - Have any been tried or adopted in the household? If so, how long does he think they will be maintained?
        - Do any of the recommended practices conflict with his own beliefs?

HH MODULE 3: SHG MEMBER RESIDENT SENIOR WOMEN

1. Knowledge and opinion of program

    a. Please ask the senior woman to tell you what he knows about the VARRAT program, being careful to distinguish between the nutrition work and the previous agriculture intervention.

        - What is her opinion of the program?
        - Has she discussed any of the nutrition topics with her daughter-in-law?
        - Does he know which videos her daughter-in-law has watched? Has her daughter-in-law shared any of the messages with her?
        - Has she shared any of the messages with anyone else?
        - What does she think of the dissemination approach?

2. Acceptance of daughter-in-law's participation in the nutrition disseminations

    a. Please explore the issue of acceptability of her daughter-in-law's participation

        - Does she have any problems with her attendance at disseminations or other activities?

3. Acceptance of new nutrition behaviors in household

    a. Explore her thoughts on the recommended practices.

        - Does she find them believable, feasible?
        - Have any been tried or adopted in the household? If so, how long does she think they will be maintained?
        - Do any of the recommended practices conflict with her own beliefs?
