# Supplementary material for: Adapting Agriculture Platforms for Nutrition: A Case Study of a Participatory, Video-Based Agricultural Extension Platform in India
Source: PLoS One. 2016 Oct 13;11(10):e0164002. doi: 10.1371/journal.pone.0164002 (PMC5063370; doi:10.1371/journal.pone.0164002)
Supplement: S2 File — (PDF) [file pone.0164002.s002.pdf]

KII: DG Staff (DGS)

Research Topic 2: Examine the capacity of VARRAT to produce MIYCN video content through the DG approach

Research Topic 3: Explore DG staff perceptions of the key factors affecting the application of the DG approach to MIYCN promotion, focusing particularly on the transition in content, as well as on constraints and challenges faced. Additionally, these interviews will examine DG staff perspectives on the most effective components of the approach as adapted to MIYCN promotion

Instructions: The researcher should elicit responses around the content areas outlined below. The researcher should look to the main points to guide conversation, in order to ensure that the relevant information is captured; however, it is important that the interviewer should avoid simply converting these subject headings into questions. The themes outlined below should be treated as 'conversation guides' that can facilitate a guided conversation between the researcher and the informant, focused on the main research themes. It is imperative that you obtain full, rich answers from the interviewee: always ask 'why?' and seek deeper explanations. Short, one-sentence answers are not sufficient. Finally, please remember that you must adjust the vocabulary according to the type of informant, while retaining the original meaning.

I. *Explore roles and responsibilities (Research Topics #2 and 3)*

a. Outline the responsibilities of the DGS:
- What is the DGS understanding of their responsibilities: to DG as their organization, to VARRAT and SPRING as partners, and to the communities they serve?
- Does the DGS feel they have the necessary support and appropriate technical preparation to undertake their responsibilities?

b. How do DGS see their own role in the program:
- What is the importance of their role as DGS?
- Does the DGS feel limited in any areas in terms of their role in the program?

c. Ask the informant to describe their involvement and relationship with VARRAT staff and SPRING:
- What are the working relationships and hierarchies between DG and VARRAT staff?
    - i. Discuss the role of VARRAT staff other than CRPs/CSPs in the pilot
- What are the working relationships and hierarchies between DG and SPRING staff?
    - i. Discuss the role of SPRING staff in the nutrition pilot
- Have any conflicts or bottlenecks over decision-making occurred during the process of implementation, and if so, what are they and why did they occur? What was done to resolve these issues?

II. *The relationship between VARRAT and communities (Research Topics #2 and 3)*

a. Ask the informant to outline interactions between VARRAT staff and community members:
- DGS' perceptions of the VARRAT staff level of familiarity with villages

- Is the posture of VARRAT staff towards the community more vertical or is it a more horizontal relationship? Does the informant have other opinions about the role VARRAT plays in the communities?
- How does the community receive the VARRAT staff in their village? Are there any tensions?
- Do community members appear to be comfortable asking questions to VARRAT staff?

b. Involvement of community in the implementation process
- Does the community participate in any way in the identification of themes, storyboarding or production of videos?
- Has the DGS received any feedback from communities on videos? If so, what contributions were made and how were they addressed? If not, are there any mechanisms by which this information could be gathered?

III. *CRP/CSP Capacity (Research Topics #2 and 3)*

a. Facilitation during video dissemination
- How comfortable are CSPs in leading facilitation for nutrition videos? In comparison with the agriculture videos?
- Are there any gender issues for CSP facilitation of nutrition videos?
- Are there any other challenges you note surrounding the video disseminations and the roles of the CSPs?

b. Adequacy of nutrition training and CSP/CRP knowledge
- Are CSPs adequately prepared in terms of nutrition concepts for video disseminations and their roles as facilitators?
- On the same note, are CRPs sufficiently prepared in nutrition technical concepts to produce videos with accuracy and a realistic quality?
- What could be done to better prepare CRPs and/or CSPs in nutrition content and knowledge? Are there specific knowledge areas that are particularly problematic for CRPS and/or CSPs to grasp? If so, what are they?

c. Capacity of CSPs/CRPS to disseminate/produce videos on their own
- How much autonomy do CSPs/CRPs have in their functions? Do they take decisions throughout the processes of production and dissemination?
- What role does the DGS play in encouraging or supporting their work? Does the DGS feel they are able to conduct these tasks without heavy supervision?
- How could any limitations be addressed?

d. Extent of external involvement
- To what extent has SPRING been involved in video production and dissemination activities?
- Is the support provided by SPRING necessary for the production of quality videos? Do CRPs appear to be increasing capacity over time (i.e., do you envision a eventual phasing-out of SPRING support), or is there still a significant knowledge or capacity gap?

IV. *Content Adaptation Process during video production (Research Topics #2 and 3)*

a) What does the DGS think about the differences between agriculture and nutrition content selection/adaptation?

- Are there any differences in the content identification and adaptation process?
- What are the differences with respect to time invested by DGS in nutrition content selection/adaptation?
- Does the informant feel that the positive deviance model is as applicable in nutrition as in agriculture?

b) What does the informant think of the videos style (local positive deviant protagonist vs. external expert)?

c) Ask the informant to describe any difficulties in content adaptation-
- Difficulties that DGS face while working with VARRAT/SPRING/community
- Difficulties that DGS face during choosing content, adapting content, producing content
- What are the unique issues in nutrition videos that are challenging in contrast with the agriculture videos?
- What are the challenges in identifying examples of positive deviance from the community?

d) Ask the informant about strengths and weaknesses of the nutrition approach.
- Do they think it is likely that SHG members will adopt and diffuse the practices?
- Did the DGS note any differences in the reception of the nutrition themes in contrast with previous agricultural-themed videos?
- What are the general community and VARRAT staff perceptions of selected themes? Is the content well received or is scepticism or questioning evident?

e) Please ask the informant to describe their own understanding of the process of theme selection for nutrition content
- Who decides the themes?
- What is the basis on which some topics are included while others are excluded?
- Did any consultation occur during this process? (SPRING, VARRAT, or communities?)

f) Has inclusion of agriculture into nutrition videos been considered?

g) Discuss the involvement of staff in the production process
- Discuss the involvement of SPRING staff in the production process
- Discuss the involvement of VARRAT staff in the production process
- Time and supervision provided by the DGS for the production process

V. *Explore the operational challenges facing the DG staff (Research Topics #2 and 3)*

a. Discuss any challenges in rolling out nutrition videos
   - Are there any problems with two screenings of agriculture being replaced by nutrition?
     - Solutions?
     -
   - Please explore the issue of partnerships (with VARRAT and SPRING). Ask the informant to tell you what is working well, and what is not working so

well, also also what kinds of solutions they are finding to address any partnership challenges.

- Ask the DGS to discuss workload and staffing. The nutrition project will have added extra work. How are staff handling this? Are the human resources sufficient?

a. .

VI. Monitoring and Assessment (Research Topics #2 and 3)

a. What is the expectation of what can be measured through COCO?
   - How is "success" measured for nutrition videos? What is the expectation of the duration and level of use of recommended practices? How does this compare with practices promoted in agriculture videos?
   - What is the opinion of the DGS about the adoption process, especially when agriculture and nutrition practices are compared?

b. Process of choosing of non-negotiable points
   - Do non-negotiable points accurately track adoption? If yes, how can this be verified? If not, are there any other mechanisms that could be utilized to track adoption or program impacts?

c. Monitoring and verification challenges:
   - DGS opinions of the greatest challenges for program M&E
   - Which practices lend themselves most easily to verifying adoption? Which practices are most challenging to verify and why?
   - What is the capacity of CSPs to conduct adoption verification process for nutrition?
   - What are the feedback loops for learning within DG? For example, if M&E identifies problems or areas of concern, what does the DGS do? How is that information received or how would it be received in DG?

VII. *Strategic Issues for Digital Green (Research Topics #2 and 3)*

a. Ask the DGS to discuss scale up. What key challenges are likely, and what conditions would be required to achieve scale up?

b. Enablers and constraints on expansion
   - What are the technical challenges on the video production side of increasing the program scale for nutrition videos?
   - What are potential issues in terms of content for scale up of nutrition videos?
   - Are there any types of nutrition content that would be appropriate in some areas, and not in others?
   - When bringing the program to scale, what kinds of conditions will need to be met?

	• Are there some components which are more amenable to scale-up than others?
