# Supplementary material for: Adapting Agriculture Platforms for Nutrition: A Case Study of a Participatory, Video-Based Agricultural Extension Platform in India
Source: PLoS One. 2016 Oct 13;11(10):e0164002. doi: 10.1371/journal.pone.0164002 (PMC5063370; doi:10.1371/journal.pone.0164002)
Supplement: S3 File — (PDF) [file pone.0164002.s003.pdf]

KII: VARRAT CRPs

Research Topic 2: Examine the capacity of VARRAT to produce MIYCN video content through the DG approach

Research Topic 4: Evaluate VARRAT staff (including CRPs and CSPs) perceptions of the strengths and weaknesses of using the DG approach to promote select MIYCN practices (deliver MIYCN messages) relative to disseminating agriculture extension training.

Instructions: The researcher should elicit responses around the content areas outlined below. The researcher should look to the main points to guide conversation, in order to ensure that the relevant information is captured; however, it is important that the interviewer should avoid simply converting these subject headings into questions. The themes outlined below should be treated as 'conversation guides' that can facilitate a guided conversation between the researcher and the informant, focused on the main research themes. It is imperative that you obtain full, rich answers from the interviewee: always ask 'why?' and seek deeper explanations. Short, one-sentence answers are not sufficient. Finally, please remember that you must adjust the vocabulary according to the type of informant, while retaining the original meaning.

I. *CRP skills and experience (Research Topic 2)*:

a. Informant motivations :
   - How did CRP get involved in the nutrition program, and what were their motivations for joining the team?

b. Explore the informant's perspectives on both technical and non-technical areas of the project.
   - Does the informant have any informal or formal background in video production or technology?
   - Does the CRP feel like he/she has sufficient preparation on the technical side? Are there any areas in which they could use more support?
   - How knowledgeable or confident does the informant feel in the area of nutrition? What is the informant's subject matter knowledge on nutrition? How does this compare to their background and/or experience in agriculture content? *NB this is also assessed with the knowledge test; here we are interested in the informant's perception of their own knowledge.*
   - Does the CRP feel they have sufficient preparation in terms of nutrition content in order to produce effective videos? If not, what sort of support is needed?
   - Explore the informant's feelings about being a CRP; the positive and negative aspects of their work.
   - Explore new responsibilities or roles as related to adapted nutrition content. How has their role changed from agriculture to nutrition production?
   - What training was helpful for the CRP in preparation for their role in producing nutrition videos? What type of training could be useful to improve their skills?

- Explore how the informant views their own role- has his work in production brought about any behavior change?

II. *Video production, content and approach (Research Topics 2 and 4)*

a. Please explore the informant's experiences with each of the following steps. Ask them to tell you about their own involvement and what their role was, and to give you their opinion on the process. Where possible, they should compare experiences with nutrition vs. agriculture video production.
   - Protagonist selection and recruitment (challenges to positive deviance model?)
   - Storyboarding
   - Filming/shooting
   - interaction with CSPs

b. In the opinion of the CRP, what are the strengths and weaknesses of using the DG approach to promote select MIYCN messages?

c. What does the CRP think about the video style (local actor vs. respected expert)- ask about the demonstration versus testimonial kind of nutrition video

d. What is the CRP's perception of the SHG members' interest and/or comfort level with the nutrition content/topics?

e. Equipment
   - Does the CRP feel that they have the sufficient and appropriate equipment to effectively produce quality videos? If not, what is lacking?
   - Are there funds to acquire what is necessary for such improvements and/or repairs?

f. Please ask the CRP to discuss the community response to the production. Does the CRP think adoption and/or diffusion are likely outcomes? Why or why not?

g. Discuss any challenges in rolling out nutrition videos
   - Are there any problems with two screenings of agriculture being replaced by nutrition?
     - Solutions?

III. *Experience with partners (Research Topic #4)*

a. Relationship with DG and SPRING in terms of content support and video development.
   - What has the relationship been like for the CRP with DG and SPRING in terms of content support on nutrition?
     - How does the CRP get nutrition information from SPRING/DG? Is this different from the way they get agriculture information?
     - What is the CRP's opinion of this channel of communication?
   - What has the relationship been like for the CRP with DG in terms of video development (presentation, storyboarding, etc.)?
     - How does the CRP get video development support from SPRING/DG? Is this different from the way they get agriculture information?
     - What is the CRP's opinion of this channel of communication?

- Discuss the dynamic between CRPs, DG and SPRING in pre-production, production and post-production/dissemination phases.

b. Decision-making:

- Does the CRP feel they have autonomy in decision-making regarding video production?
- If not, who generally in what way makes decisions in regards to content selection, filming techniques and presentation style?

c. Relationship with existing community-based health and nutrition interventions

- Ask the informant to discuss their opinion of the role of AWWs and other health staff in the program. Are synergies being created? Is the relationship positive or negative?

IV. Content Development (Research Topic #4)

a. Perspectives on the transition to new nutrition content.

- How has informant felt about the transition to the new nutrition content?
- Has the new content changed the amount of work and responsibilities of CRPs?

b. Primary production challenges, both technical and non-technical, to adapt to new content. Please discuss with reference to each of the nutrition videos.

- Are there unique challenges for the visual representation for nutrition content from a technical standpoint (in filming, storyboarding problems like multiple actors, lighting, sound issues)?
- Are there any conceptual challenges for representation of nutrition content? (i.e. showing nutrition concepts such as 1000 days)
- Are there any socio-cultural challenges for representation of nutrition content?
  (i.e. gender and caste of protagonists, taboo topics)
- Are there any other challenges or difficulties that have emerged during the production of nutrition videos so far?
