# Supplementary material for: Adapting Agriculture Platforms for Nutrition: A Case Study of a Participatory, Video-Based Agricultural Extension Platform in India
Source: PLoS One. 2016 Oct 13;11(10):e0164002. doi: 10.1371/journal.pone.0164002 (PMC5063370; doi:10.1371/journal.pone.0164002)
Supplement: S4 File — (PDF) [file pone.0164002.s004.pdf]

KII: VARRAT CSPs

Instructions: The researcher should elicit responses around the content areas outlined below. The researcher should look to the main points to guide conversation, in order to ensure that the relevant information is captured; however, it is important that the interviewer should avoid simply converting these subject headings into questions. The themes outlined below should be treated as 'conversation guides that can facilitate a guided conversation between the researcher and the informant, focused on the main research themes. It is imperative that you obtain full, rich answers from the interviewee: always ask 'why?' and seek deeper explanations. Short, one-sentence answers are not sufficient. Finally, please remember that you must adjust the vocabulary according to the type of informant, while retaining the original meaning.

I. *CSP skills and experience (Research Topic 1):*

a. Informant motivations
   - How did CSP initially get involved in the program, and what were their motivations for joining the team?

b. Explore the informant's perspective on both technical and non-technical areas of the project
   - Does the informant have any informal or formal background in facilitation or in
     the content covered by the nutrition videos? (For instance, is the CSP a health worker?) Do they have an informal or formal background in agriculture?
   - How prepared do they feel to carry out the nutrition dissemination? Do they feel they have sufficient preparation on the technical side about nutrition topics to disseminate? If not, what is lacking and how do they need support?
   - Ask about the informant's feelings about being a CSP; the positive and negative aspects of their work .
   - Ask about new responsibilities or roles as related to adapted nutrition content. How has their role changed from agriculture to nutrition dissemination?
   - What training was helpful for the CSP in preparation for their role as facilitating nutrition videos? What type of continued or additional training could be useful to improve their skills?
   - Ask about how the informant views their own role – has her work in dissemination brought about any behavior change?

II. Video dissemination, content and approach (Research Topic 4)

a. Please explore the CSP's experiences with each of the following steps. Ask them to tell you about their own involvement and what their role was, and to give you their opinion on the process. Where possible, they should compare experiences with nutrition vs. agriculture video dissemination. Also, whenever possible, please try to make reference to specific videos rather than general options.

- Content
    - Their opinion of the content selected for nutrition video disseminations.
    - Does the CSP feel that the content is acceptable, feasible and appropriate for SHG members and the larger community?
- Target Audience
    - Please ask about CSP's views on who the target audience should be- only SHGs or others as well? Husbands? Why?
    - What is the CSP's perception of the SHG members' interest and/or comfort level with the nutrition content/topics?
- Acceptance
    - Were there any particular videos that the community preferred or appeared to enjoy or engage with the most? Why do you think those videos were preferred above others?
    - Were there any particular videos that generated discussion, debate or conflict? If so, which videos, and why does the informant think they were cause for such dialogue?
    - Their perspectives on video style and production- ask about demonstration versus testimonial; their perspectives on credibility, feasibility.
- Facilitation
    - Challenges faced in facilitation (use local term for this please) of nutrition content- nutrition knowledge of CSP, dealing with conflict topics, reaction of women, questions asked, participation level of audience
    - When a video generates debate, what do you do?

b. Ask the CSP about how taboos are dealt with in nutrition videos and disseminations. Explain carefully what you mean by taboo.

- What are local taboos on issues of nutrition in the intervention communities? Are these taboos problematic? If so, which ones, and why?
- Are there certain stakeholders or actors who perpetuate taboos more than others?
- Has there been any effort to engage these local actors in receiving exposure to nutrition content? Are these actors open to the nutrition messages being shown by DG?
- Is there any effort by local actors to contradict or discredit the content in DG videos?

- Does the content selected talk about or mention local taboos? If so, which taboos were deemed most important to address and how were they addressed? If not, why were certain taboos not addressed?
- Who are those decision-makers in the household who can be targeted to address taboos? Do these decision-makers participate in video disseminations?

c. Ask the CSP if the nutrition videos have brought about behavior change in her household and whether she has spread the message to others? Why? Why not?
d. Ask CSP if she thinks the DG model (remember to explain DG Model carefully in local terms) is the right approach for nutrition dissemination and whether she thinks it's different from the traditional Behavior Change Communication messages that come from the health worker. What are the differences that she feels are present?
e. Does the CSP have opinions about the strengths and weaknesses of this nutrition project?
f. Please ask the CSP to discuss the community response to the videos and disseminations. Does the CSP think adoption and/or diffusion are likely outcomes? Why or why not?
g. Discuss any challenges in disseminating out nutrition videos
    - Are there any problems with some of the agriculture screenings being replaced by nutrition?
        - Solutions?

IV. Adoption check process (Research Topics 1 and 4)

a. Please ask the CSP to tell you what they know about the adoption checking process for nutrition practices, and about their role in it.
b. If they are able to describe the process, ask them to tell you their opinion of it
c. Ask the CSP to compare the strengths and weaknesses of adoption checking in agriculture vs. nutrition practices.

V. Relationship with community (Research Topics 7 and 8)

a. Please discuss the following topics with the CSP, again, ensuring that you raise differences between nutrition and agriculture dissemination work:
    - How the CSP feels s/he is received in villages.
    - Whether attendance at the dissemination sessions is the same, greater, or lesser than for agriculture sessions. Her opinion on why this might be the case.
    - How well the new content is received by session attendees. Are they keen to learn? Hostile? Embarrassed or ashamed? Enthusiastic?
    - Do SHG members seem to understand the content presented in the videos? Do they ask questions?
    - Do SHG members discuss content outside of the sessions, with other participants or with other community members?
        a. If so, in what capacity is it discussed, where and with whom? How do SHG members talk about the sessions or content? Can you give an example?
    - Does the informant feel that some messages are more likely to be adopted than others? If so, which ones and why?

- Has there been any demand for video disseminations to be repeated, or to be shown in other locations or communities? If so, where and why?
- Do other community members who did not participate in the video sessions show interest or ask about the video sessions? Is there any reason they do not attend?

b. Relationship with existing community-based health and nutrition interventions

- Ask the informant to discuss their opinion of the role of AWWs and other health staff in this program. Are connections being made? Is the relationship positive or negative?

V. Relationship with partners (Research Topic 4)

a) Ask about the dynamic between CSP and DG staff.

- Are there any new areas of contention or discussion in either the responsibilities or behavior of CSP (facilitation, technical capacity, etc.) or content with the addition of nutrition disseminations?
- Does the CSP feel well-supported by VARRAT in their work?
- Do they have time to do everything?
- Is there any way they could be better supported in the work they do?
- What are some of the challenges the CSP faces in carrying out their nutrition disseminations? Solutions?

VI. Issues of scale (Research Topic 4)

a. Enablers and constraints on expansion

- What are the technical challenges on the video dissemination side of increasing the program scale for nutrition videos?
- What are potential issues in terms of content for scale up of nutrition videos?- For instance, if dissemination expands from the current 30 villages to more villages?
- Are there any types of nutrition content that would be appropriate in some areas, and not in others? Or during some seasons and not in others?
