# Supplementary material for: Adapting Agriculture Platforms for Nutrition: A Case Study of a Participatory, Video-Based Agricultural Extension Platform in India
Source: PLoS One. 2016 Oct 13;11(10):e0164002. doi: 10.1371/journal.pone.0164002 (PMC5063370; doi:10.1371/journal.pone.0164002)
Supplement: S5 File — (PDF) [file pone.0164002.s005.pdf]

KII: AWW/Health Workers

Research Topic 1: Examine the capacity of the VARRAT CSPs to deliver MIYCN messages through the DG approach as well as determinants of differential capacity among CSPs, and across content areas in nutrition

Research Topic 2: Examine the capacity of VARRAT to produce MIYCN video content through the DG approach

Research Topic 5: Assess the acceptance by self-help group members of the DG approach for delivery of MIYCN messages as well as perceptions of the strengths and weaknesses of using the approach

Research Topic 8: Understand intra-household/ -community diffusion effects, if any, of the intervention on the knowledge and attitudes of households that do not participate in video disseminations as well as on community health and nutrition workers.

Instructions: The researcher should elicit responses around the content areas outlined below. The researcher should look to the main points to guide conversation, in order to ensure that the relevant information is captured; however, it is important that the interviewer should avoid simply converting these subject headings into questions. The themes outlined below should be treated as 'conversation guides' that can facilitate a guided conversation between the researcher and the informant, focused on the main research themes. It is imperative that you obtain full, rich answers from the interviewee: always ask 'why?' and seek deeper explanations. Short, one-sentence answers are not sufficient. Finally, please remember that you must adjust the vocabulary according to the type of informant, while retaining the original meaning.

I. Introductory questions

a. Please ask the informant if she has heard of the DG intervention (using correct local term for this).
b. Is the informant a member of the SHG?
c. Has the informant attended any agriculture video disseminations?
d. Has the informant attended any nutrition video disseminations?
    - If so, which ones?
    - Was there any she liked best? Why?
    - Were there any she did not like? Why?
    - If she has not seen any nutrition videos, please explore what the reasons were and then end the interview. Inform supervisor who will identify a different informant.

II. Perceptions of DG model and content (Research Topics 1, 2, 5)

a. Effectiveness of nutrition videos especially in comparison to agriculture *videos*
- What are the informant's opinions on the topics in the DG video project
- Ask the informant if she thinks there is potential for the videos to bring about behaviour change.

- Ask the informant about her experience with standard behaviour change communications done through their ASHA/AWW/ANM work and how the DG nutrition approach is different.
- Which approach does she think is more effective
- Ask the informant to tell you what differences she sees between using the approach for agriculture, and using it for nutrition.

b. Perceptions of nutrition model in aiding informant work
    - Does the informant think that the video messages reinforce or support the health promotion work that the informant has been doing or knows of? If yes, in what ways? If no, why not?
    - What is her opinion of videos that have Discuss the positive deviance model contrasted with the demonstration model videos to find out which the informant considers a more effective method. For instance, someone in the village who has used a good health practice talking about its benefits in the video versus a lecture being given by a health expert in the video.
    - Are there any conflicts between the messages of the videos and the messages offered by the informant.

c. Acceptance of messages by communities
    - Has the informant seen the SHG members discussing the video messages outside of the dissemination context? Where?
    - Are there any videos which prompted more discussion than others? Which ones? Why does the informant think this was the case? Probe for reasons.
    - Were there any videos which the informant thinks the community liked more? Or disliked? Why? Probe for reasons.
    - Have the SHG members who watch the nutrition videos requested any new information or asked the informant any questions/clarifications?
    - Discuss whether the informant thinks that the messages are acceptable to the community. Do any of the topics present problems, cause embarrassment or shame?
    - Ask the informant if she thinks the messages in the videos can be easily practiced in the community.
    - Capability of CSPs/CRPs
    - Is the informant involved in any way with the production of videos? Inputs? Acting?
    - Is the informant involved in any way with the dissemination of the videos? Is she a CSP or does she assist the CSP in any way during dissemination?
    - What is the informant’s opinion about the production of the nutrition videos and their quality in terms of content, protagonist selection, authenticity and feasibility?
    - What is the informant’s opinion about the disseminations of nutrition videos? CSP’s capabilities? SHG members’ involvement and discussions?
    - Ask the informant if she thinks the CRPs and CSPs face any particular challenges in producing and disseminating the videos (such as lack of formal nutrition training, increased workload).

d. Capability of CSPs/CRPs (use local term so healthworker understands)
   - Is the informant involved in any way with the production of videos? Inputs? Acting?
   - Is the informant involved in any way with the dissemination of the videos? Is she a CSP or does she assist the CSP in any way during dissemination?
   - What is the informant's opinion about the production of the nutrition videos and their quality in terms of content, protagonist selection, authenticity and feasibility?
   - What is the informant's opinion about the disseminations of nutrition videos? CSP's capabilities? SHG members' involvement and discussions?
   - Ask the informant if she thinks the CRPs and CSPs face any particular challenges in producing and disseminating the videos (such as lack of formal nutrition training, increased workload).

III. *Relationship with DG/VARRAT (Research Topic 5)*

a. Interaction with DG/VARRAT staff; any involvement in program
   - Discuss any informants involvement in any of the production or dissemination activities of the project.
   - Ask the informant to tell you about any contact she has had with DG or VARRAT staff, related to this project.

b. Assistance provided to VARRAT during production/dissemination
   - Does the informant offer any help in disseminations for clarifying questions or mediation?
   - Ask the informant if she believes she has a role to play in the DG project, and if she does, then ask her to tell you about her perception of this role.

IV. Community Dynamics and change (Research Topic 8)

a. Reaching Targets
   - Does the informant think that the videos are reaching out to pregnant women, mothers of undernourished children, adolescent girls and mothers-in-law? Ask about how many women in VHNDs know about these messages (pregnant, lactating and mothers of young children) and ask about adolescent girl meetings and other meetings she holds with women in the village.
   - Does the informant think that SHG members share information from disseminations with other community members who did not attend?

b. Regularity of Anganwadi visits or check-ups by community members (only ask these of the AWW, not of ASHA or ANM)
   - Is there an Anganwadi Centre in the village and how active is it?
   - How regularly do the community members avail the services of the Anganwadi centre?

- Has this number increased since the DG project in nutrition began? Why or why not? Look at the attendance data.
- Does the informant feel that her own role in the community has changed at all?
- Ask the informant if the community views her as an expert in topics where (1) she has acted in videos (2) she has not acted in the videos?

c. Nutrition messages and key decision makers in the village and home
    - How are the nutrition messages reaching the key decision makers in the homes?
    - Ask the informant if she sees any major challenges to this model of MIYCN messaging through DG? If yes, what and why? Solutions?
