# Supplementary material for: Adapting Agriculture Platforms for Nutrition: A Case Study of a Participatory, Video-Based Agricultural Extension Platform in India
Source: PLoS One. 2016 Oct 13;11(10):e0164002. doi: 10.1371/journal.pone.0164002 (PMC5063370; doi:10.1371/journal.pone.0164002)
Supplement: S6 File — (PDF) [file pone.0164002.s006.pdf]

KII: VIDEO PROTAGONIST

Research Topic 2: Examine the capacity of VARRAT to produce MIYCN video content through the DG approach

Research Topic 4: Evaluate implementers' perceptions of the strengths and weaknesses of using the DG approach to deliver MIYCN messages relative to disseminating agriculture extension training

Research Topic 8: Understand intra-household /-community diffusion effects, if any, of the intervention on the knowledge and attitudes of households that do not participate in video dissemination as well as on community health and nutrition workers.

Instructions: The researcher should elicit responses around the content areas outlined below. The researcher should look to the main points to guide conversation, in order to ensure that the relevant information is captured; however, it is important that the interviewer should avoid simply converting these subject headings into questions. The themes outlined below should be treated as 'conversation guides' that can facilitate a guided conversation between the researcher and the informant, focused on the main research themes. It is imperative that you obtain full, rich answers from the interviewee: always ask 'why?' and seek deeper explanations. Short, one-sentence answers are not sufficient. Finally, please remember that you must adjust the vocabulary according to the type of informant, while retaining the original meaning.

**I.**	*Recruitment*<br>a. What is the protagonist's understanding of why they were selected (under what criteria do they believe they were recruited)? Was this explained to them?<br>b. Did the protagonist fully understand what their role would be when they were selected?<br>c. How did the protagonist feel about being selected to participate in video production?<br>d. Has the protagonist watched any videos, including their own? Which ones?<br>e. Is the protagonist an SHG member?
**II.**	*Production process*<br>a. Experience during production process<br>i. How comfortable did the protagonist feel in their role as protagonist in the videos?<br>b. Previous experience with production<br>ii. Did the protagonist have any previous experience participating in the agriculture videos? If so, how was the experience different for agriculture versus nutrition?
**III.**	Challenges during production process<br>a. Was the production process easy or hard for the protagonist?<br>b. What are the main challenges to the production process that the protagonist faced? [*these may be of any type, whether in the protagonist responsibilities, in comprehension of messages, etc. – let the protagonist define what they saw as being a challenge rather than leading them to a specific type of answer*]

c. Explore if they have solutions for these challenges
d. What are the main challenges to video production in general, in the eyes of the protagonist?
e. Explore if they have solutions for these challenges

IV. Knowledge gain

i. Does the protagonist feel they have gained any nutrition knowledge during the production process of the video?
ii. Which are those concepts and practices they particularly remember?
iii. Has the protagonist tested any of the behaviors or practices in their household? Why or Why not
iv. Did the protagonist continue to use the recommended behavior or practice? Why or why not?

V. If she has adopted the practice, please explore:

i. If she adapted the practice to her reality/local setting (i.e. substituting cheaper soap, etc.) to make it feasible;
ii. What value does the interviewee place on the information shared? Do they find this information useful in their daily life?
iii. Whether she thinks the practice is effective and why;
iv. Whether she finds the practice easy, and why (please ensure that you cover the full range, from practical issues through to socio-cultural reasons);
v. Whether she finds it difficult, and why (please ensure that you cover the full gamut, from pragmatic issues through to socio-cultural logic);
vi. Whether or not other household members are supportive of the adoption – and why or why not;
vii. How long she thinks she will be able to continue doing the practice.

VI. *Perceptions of video dissemination approach*

a. Protagonist opinion on videos
   - How does the protagonist feel about the choice of content on nutrition issues for video disseminations?
   - What is the protagonist's opinion on the presentation style of nutrition issues in the videos? Probes:
     - Was the story or script interesting?
     - Who in the video was targeted in the video?

- Who in the video was giving the information?
- Which family or community members were included in the video?
- Was it easy to understand? Was it interesting?

- Do they see ways in which the presentation of the content could be improved or made easier for the audience to understand?

b. Opinions of DG model for nutrition

- What are the protagonist's thoughts on the DG model for nutrition video dissemination (explain DG model to the interviewee carefully)? Are they familiar with the agriculture videos? If so, what do they see as the main differences between the two models, particularly in terms of the role of protagonists and the acceptance of messages in the community?

VII. *Self-perceptions*

a. Reasons for participation in video production

- What are the protagonist's motivations for getting involved with video production?

b. Role as protagonist

- Does she feel that she is now a role model/expert/leader for this behaviour or feel responsible to encourage others to adopt it?

VIII. *Role in the community and diffusion*

a. Changes in relationships in community

- Has the protagonist perceived any changes in their relationships with members of their village/community? Do people receive him/her any differently? If so, how?
- How does the protagonist feel about these changes?

b. Challenges of new role as protagonist

- In their new role as protagonist, have any unique situations have emerged?

c. Please ask the protagonist whether she has shared any information, knowledge or experience learned in the nutrition video production with anyone, either within or outside her household.

- If not, ask her why she hasn't shared the information. Probes:
    - Lack of opportunity
    - Lack of interest
    - Other reasons (describe)
- If she has shared information, knowledge or experience with anyone in her social network (including family), please ask:
    - What she has shared;
    - With whom she has shared it;

	• Why she decided the share the information, knowledge or experience.
