# Supplementary material for: Adapting Agriculture Platforms for Nutrition: A Case Study of a Participatory, Video-Based Agricultural Extension Platform in India
Source: PLoS One. 2016 Oct 13;11(10):e0164002. doi: 10.1371/journal.pone.0164002 (PMC5063370; doi:10.1371/journal.pone.0164002)
Supplement: S7 File — (PDF) [file pone.0164002.s007.pdf]

Nutrition Knowledge Test:

Name of the Village: ______________________

Index SHG member ID: _______________________

Full Name of the Index SHG Member/Source of information: _____________________

Date of the interview: ____________________________

Instructions: This nutrition knowledge test should be applied to index SHG member, all CSPs, and all CRPs. This instrument is distinctly different from the Key informant interviews and social network analysis in that it is intended to test informants on specific topics that were disseminated in video sessions. It is extremely important for the researcher to avoid influencing the response of the informant, whether directly or indirectly (facial expressions, etc. may affect the answer choice by informants, for example).

1. **What is nutrition and why is it important?**

2. **What is the most important period for improving child nutrition: (circle all mentioned)**

   a. First 1000 days ….1
   b. Pregnancy…….2
   c. Lactation………3
   d. 0-6 months……4
   e. 6-24 months……5
   f. 24-59 months……6
   g. Over 5 years……..7
   h. Adolescent girls……8
   i. Adolescent boys……9

3. **Have you heard of first 1000 days?** (ask if the person does not mention *option a.* above)
   Yes….1
   No ….2>>**Skip to 5**

4. **Explain what you heard about first 1000 days**. If the respondent mentions the *response in italics* below, check the box.

   ☐ *The first 1000 days is important because this is the time when most physical and mental development of the baby happens.*

S.N.	Question	Response
5	**When do you believe that a young child can be given the following liquids and foods? How old should the child be?**	
5.1	Water or other clear liquids<br>(e.g. honey, broth, juices ,etc.)	Months.......................................1 ☐☐<br>Weeks ....2 ☐
5.2	Milk or other than breast milk<br>(e.g. tinned, powdered, or fresh animal milk or dairy products, such as yogurt, etc.)	Months.......................................1 ☐☐<br>Weeks ....2 ☐
5.3	Semi-solid foods (e.g. Lito, Jaulo, Khichadi, etc.)	Months.......................................1 ☐☐<br>Weeks ....2 ☐
5.4	Solid foods (e.g. Rice, Vegetable, Roti, etc.)	Months.......................................1 ☐☐<br>Weeks ....2 ☐
5.5	Eggs	Months.......................................1 ☐☐<br>Weeks ....2 ☐
5.6	Animal meat/Fish (e.g. chicken, duck, goat, lamb, buffalo, wild boar, pig, fish, etc.)	Months.......................................1 ☐☐<br>Weeks ....2 ☐
6	When should a baby first be breastfed?	Immediately after birth ....1<br>With in 1 hour..2<br>After 1 hour but within 1 day ....3<br>After 1 day but within 2 days...................4<br>After 2 days...5<br>Baby should never be breastfed ....6<br>Other (Specify)__________________ .....96<br>Don't know..98
7	What should a mother do with the “first yellowish milk” or colostrum?	Throw it away ....1<br>Give it to her baby ....2<br>Other (Specify) ______________ ....96<br>Don’t know/remember..........................98
8	If a mother thinks she does not have enough breast milk, what should she do?<br><br>**(Multiple answers possible. Don't read possible answers)**	Breastfeed more often/more frequently. 1<br>Breastfeed the child on demand ....2<br>Give other liquids/foods ....3<br>After emptying one breast, switch to the other ....4<br>Feed animal milk to the child...................5<br>Mother needs to drink more water/liquid6<br>Mother needs to eat more food ....7<br>Mother needs to eat more nutritious food..8<br>Stop breastfeeding...................................9<br>Other (Specify)__________________ .....96<br>Don’t know 98
9	How often should a mother breastfeed her baby? **(Check only <u>one</u> answer)**	On demand both night and day…….1<br>On demand in the morning………2<br>On demand at night only…………3<br>Feed only at a predetermined schedule…4<br>Other, explain………….5

S.N.	Question	Response
10	What should you do in relation to feeding a child to recover from illnesses?<br><br>**(Multiple answers possible. Don't read possible answers*)***	Feed an extra meal daily......................1<br>ORS ....2<br>Feed less food than usual ....3<br>Feed as much food as usual.................4<br>Feed more food than usual ....5<br>Feed different types of foods ....6<br>Give less liquids than usual..................7<br>Give as much liquid as usual................8<br>Give more liquids than usual ....9<br>Give different types of liquid than usual...10<br>Increase frequency of breastfeeding. 11<br>Stop breastfeeding ....12<br>Give safe drinking/treated water ....13<br>Give carrot juice or rice scum ....14<br>Give Zinc tablets ....15<br>Continue breastfeeding ....16<br>Give syrups ....17<br>Give traditional medicine ....18<br>Go to health facility ....19<br>Other (Specify)_______________ ......96<br>Don't know ....98<br>**If 2-98 go to Q12**
11	For how long should you feed an extra meal daily? **(Ask only if the answer to above is 1)**	No. of days.................................☐☐
12	During the whole pregnancy, how many times should a woman go for ANC checkups?	Number of times ☐☐
13	During the whole pregnancy, how many times should a woman receive a TT injection?	Number of times ☐☐☐
14	Do you think there are any benefits to taking IFA IFA tablets during pregnancy? What are they?<br><br>(Multiple answers apply)	Mother will be healthy ………………….1<br>Mother will give birth a healthy child…….2<br>Baby will not be prematurely born ……3<br>Will not be of low birth weight…..4<br>Others, specify……5<br>None…..6
15	When should pregnant women take start IFA tablets? And how frequently? **(Check only <u>one</u> answer)**	Everyday starting first trimester……1<br>Weekly starting first trimester….2<br>Once in two weeks starting first trimester……………………………….3<br>Everyday starting second trimester……4<br>Weekly starting second trimester….5<br>Once in two weeks starting second trimester…………………………….6<br>Everyday starting third trimester……7<br>Weekly starting third trimester….8<br>Once in two weeks starting third trimester……………………………………….9<br>Never heard about IFA……………………10

		Other, specify:…………………………….11<br>**If 10, go to Q22**
16	During the whole pregnancy, for how many days should a woman take iron/folic acid tablets?	Number of days
17	During the post partum-period, for how many days should a woman take iron/folic acid tablets?	Number of days ……………………….<br>Should not take…………………….1
19	Do you think adolescent girls should be taking IFA?	Yes….1<br>No…..**2**
20	What is the age group of adolescents that should be taking IFA	Age ----------to age --------------
21	What are some of the ways to prevent side effects from taking IFA tablets?	Take IFA tablets after the evening meal…..1<br>Do not take IFA on an empty stomach……2<br>Drink extra water…….3<br>Eat good amount of green leafy vegetables and fruits…4<br>Others, specify………5<br>Do not know…98
22	When should you wash your hands?<br><br>**(Multiple answers possible. Don't read possible answers)**	After defecation .... 1<br>After cleaning the child's bottom .... 2<br>Before preparing food/cooking .... 3<br>After cooking .... 4<br>Before eating .... 5<br>Before feeding the child .... 6<br>After feeding the child .... 7<br>After cleaning the house/compound....... 8<br>After disposing garbage .... 9<br>Before picking up the child .... 10<br>Other (Specify)______________ .... 96<br>Don't know 98
23	Would you show me exactly what you do when you usually wash your hands? (Record all the steps the mother shows you that she does to wash her hands.)<br><br>**(Please circle all that apply.)**<br><br>**(Try to get her to go to the hand washing spot so you can observe the actual practice.)**	Uses running water.............................1<br>Uses clean water including from a pot..2<br>Uses soap/ash......................................3<br>Rubs hands together only once ....4<br>Rubs hands together at least 3 times......5<br>Scrub in between fingers, under nails, and backs of only 1 hand…………………………..6<br>Scrub in between fingers, under nails, and backs of both hands……………………….7<br>Washes both hands ....8<br>Dries hands hygenically, by air or by using a clean cloth 9
24	When pregnant, should a woman eat less than usual, about the same amount as usual, or more than usual? **Probe if answer is 3. And check mark if the answer below is mentioned**	Less than usual ....1<br>Same as usual ....2<br>More than as usual ....3<br>Don't know 98

24	☐ …… One meal extra for pregnant women each day	
25	What will happen if a woman is not eating properly during pregnancy?	Baby will be prematurely born ….1<br><br>Baby will be low birth weight…….2<br><br>Difficult labor…………………………….3<br><br>Other, explain………………………….4
26	When a woman is lactating should she eat less than usual, about the same amount as usual, or more than usual?<br>**Probe if answer is 3. And check mark if the answer below is mentioned**	Less than usual ....1<br>Same as usual ....2<br>More than as usual ....3<br>Don't know……………………………………98
26	☐ Two extra meals/day for lactating women	
27	What is considered a healthy diet for pregnant and lactating women?	Green leafy vegetables and other vegetables…..1<br>Seasonal Ripe fruits…..2<br>2 to 3 times per week Animal source foods like eggs, fish and meat….3<br>Others, specify……………….4
28	I will read the following statements. Tell me whether you agree or disagree and why? **(Read each statement and circle either agree or disagree)**<br>Pregnant women need to rest to help her body save energy for labor……………………….**Agree/disagree**<br><br>Pregnant women need to work more for easy delivery………… **Agree/disagree**<br><br>Working hard up till the delivery will keep the woman strong for labor……………… **Agree/disagree**<br><br>Rest during pregnancy leads to easier delivery……..**Agree/disagree**<br><br>Rest during pregnancy will make it difficult for woman to give birth…………….**Agree/disagree**<br><br>Strenuous work in the last trimester leads to small babies and therefore easy delivery**…………** **Agree/disagree**<br>strenuous work in the last trimester can result in baby being born too early (premature baby)…….. **Agree/disagree**<br><br>Strenuous work in the last trimester can result in baby being born underweight (low birth weight)……………………………………………………………… **Agree/disagree**	

29	How can a working mother exclusively breastfeed her baby?<br><br>(Circle all that apply)	Breastfeed more often/more frequently when mother is at home……………………………………………………..……..1<br>Give milk substitute/ other liquids/foods……..2<br>Feed from bottle…………………………………………..….3<br>Express breast milk……………………………………….4<br>Other (specify)……………………………………………….5
30	How can the family of a working mother support in the exclusive breastfeeding of her baby?<br><br>(Circle all that apply)	Share the workload of the working mother…………1<br>Help to carry baby to the workplace for feeding….2<br>Accompany mother to the field with baby and take care of baby while mother works…………………………………3<br>Others (specify)…………………………………………………..4
