# Supplementary material for: Adapting Agriculture Platforms for Nutrition: A Case Study of a Participatory, Video-Based Agricultural Extension Platform in India
Source: PLoS One. 2016 Oct 13;11(10):e0164002. doi: 10.1371/journal.pone.0164002 (PMC5063370; doi:10.1371/journal.pone.0164002)
Supplement: S8 File — (PDF) [file pone.0164002.s008.pdf]

SNA mapping

Name of the Village: ______________________

Index SHG member ID: _______________________

Full Name of the Index SHG Member/Source of information: _____________________

SNA administered to (tick the relevant SNA degree)- 1st Degree/ 2nd Degree

Video No. 1 - Benefits of Hand washing with Soap

**S.NO**	**Question**	**Response**
**1**	Has anyone in your community shared information on the benefits of **hand washing** with you in the last 3 months?	**Yes…1**<br>**No…2 If NO SKIP to 2**
**1.1**	Who /what is the source of this information?	Index SHG member…**1**<br>AWW…**2**<br>AHSA….**3**<br>ANM…**4**<br>Doctor…..**5**<br>Others within the HH: **6**<br>Others outside the HH<br>(if not index SHG member)…**7**<br>Radio….**8**<br>TV……**9**<br>Newspaper…..**10**<br>Others, specify:______

**1.2**	What specific message about hand washing did [Name of SHG member] share with you in the last 3 months? And how many times<br><br>*Encourage the respondent to talk about each message and what was shared*	Explained how to wash hands………………………**1** **Times** ☐<br><br>When should people wash their hands ………**2** **Times** ☐<br><br>The benefit of hand washing with soap?........**3** **Times** ☐
**1.3**	When [NAME] shared information with you about **how to wash hands** what specific practices were mentioned<br><br>*Check all that apply and write the detailed response in the text box*	Use soap …..1<br>Rinse with clean AND running water (tippy tap or mug and bucket for pouring water)…..2<br>Rub palms together for lather…..3<br>Scrub in between fingers, under nails, and backs of hands……………………………………………4<br>Dry with clean cloth or air dry………5<br>Create a hand washing stations near the kitchen, latrine ..5<br>Other, explain……..6
**1.4**	When [NAME] shared information with you about **when should people wash their hands what specific times for hand washing was mentioned?**<br><br>*Check all that apply*	Before preparing food…**1**<br><br>Before eating …**2**<br><br>Before feeding baby ….**3**<br><br>Other, explain ….**4**
**1.5**	What did [NAME] convey about **the benefit of hand washing**<br><br>*Check all that apply*	Prevents infections**…1**<br>**OTHER CODES…..2**

**1.6**	Did you completely understand the message when it was shared to you?	Yes, completely understood the entire message/practice that was shared…..1<br>2-No, did not understand first but asked questions which the informant answered……2<br>3-No, did not understand even after asking questions because informant could not answer……3<br>4-No, did not understand and did not ask questions…4
**1.7**	Have you tested any of the recommended behaviors that were shared to you?<br>**if tested, skip to 1.9**	Haven't tested…..**1**<br>Use soap …..2<br>Rinse with clean AND running water (tippy tap or mug and bucket for pouring water)…..3<br>Rub palms together for lather…..4<br>Scrub in between fingers, under nails, and backs of hands…………………………………………..5<br>Dry with clean cloth or air dry………**6**<br>Create a hand washing stations near the kitchen, latrine..7<br>Washed hands Before preparing food…**8**<br>Washed hands Before eating …**9**<br>Washed hands Before feeding baby ….**10**
**1.8**	If you have not tested the behavior/practice, why not?<br>**if not tested skip to 1.11**	Did not think important….1<br>Lack of opportunity……2<br>Thought it was too complex….3<br>Did not understand the information well….4<br>Resource constraints….5<br>Lack of family support…6<br>Other, explain…7
**1.9**	Which of the practices did you adopt consistently	Haven't adopted…..**1**<br>Use soap …..2<br>Rinse with clean AND running water (tippy tap or mug and bucket for pouring water)…..3<br>Rub palms together for lather…..4<br>Scrub in between fingers, under nails, and backs of hands…………………………………………..5<br>Dry with clean cloth or air dry………**6**<br>Create a hand washing stations near the kitchen, latrine ..7<br>Washed hands Before preparing food…**8**

		Washed hands Before eating …**9**<br><br>Washed hands Before feeding baby ….**10**
**1.10**	IF you have not adopted the behavior/practice, why not?	Did not think important….1<br>Lack of opportunity……2<br>Thought it was too complex….3<br>Did not understand the information well….4<br>Resource constraints….5<br>Lack of family support…6<br>Other, explain…7
**1.11**	Have you shared the messages you heard about hand washing practices with any one	Yes…..1, **if yes, skip to 1.13**<br>NO…..2
**1.12**	If no, why? (note the qualitative response in the box)	Did not think important….1<br>Lack of opportunity……2<br>Thought it was too complex….3<br>Did not understand the information well….4<br>Did not think people will adopt….5<br>Other, explain…6

**1.13**	What specific practices did you recommend? And to whom (note the details of the person to follow-up with)	Use soap …..2<br>Rinse with clean AND running water (tippy tap or mug and bucket for pouring water)…..3<br>Rub palms together for lather…..4<br>Scrub in between fingers, under nails, and backs of hands…………………………………………….5<br>Dry with clean cloth or air dry………**6**<br>Create a hand washing stations near the kitchen, latrine ..7<br>Washed hands Before preparing food…**8**<br><br>Washed hands Before eating …**9**<br><br>Washed hands Before feeding baby ….**10**	**WHOM and relationship (for follow up)**

Video No. 2- Importance of first 1000 days

S.NO	Question	Response
**2**	Has anyone in your community shared information on the **Importance of first 1000 days** with you in the last 3 months?	**Yes…1**<br>**No…2 If NO SKIP to 3**
**2.1**	Who /what is the source of this information?	Index SHG member…**1**<br>AWW…**2**<br>AHSA….**3**<br>ANM…**4**<br>Doctor…..**5**<br>Others within the HH: **6**<br>Others outside the HH<br>(if not index SHG member)…**7**<br>Radio….**8**<br>TV……**9**<br>Newspaper…..**10**<br>Others, specify:______
**2.2**	What specific message about the Importance of first 1000 days video did [Name of SHG member] share with you in the last 3 months? And how many times	Pregnant woman should eat an extra meal a day……………………………………**1 Times** ☐<br>Breastfeeding mothers should eat 2 extra meals a day……………………………**2 Times** ☐<br>Institutional delivery…………………………………………………………………………**3 Times** ☐<br>Initiate breastfeeding during first hour of birth…………………………………………**4 Times** ☐<br>Exclusive breastfeeding in the first 6 months………………………………………………**5 Times** ☐<br>Introduction of Complementary feeding along with breastfeeding from 6th month to the 24th months…………………………………………………………………………………………**6 Times** ☐

**2.3**	What did [NAME] convey about **the importance of first 1000 days**	Important for physical and mental development of the baby**…1**<br>**OTHER CODES……………2**
**2.4**	Did you completely understand the message when it was shared to you?	Yes, completely understood the entire message/practice that was shared…..1<br>2-No, did not understand first but asked questions which the informant answered……2<br>3-No, did not understand even after asking questions because informant could not answer……3<br>4-No, did not understand and did not ask questions…4
**2.5**	Have you tested any of the recommended behaviors that were shared to you?<br>**If respondent not in first 1000day period/relevant case, skip to 2.9**<br>**If tested, skip 2.7**	Haven't tested…..**1**<br><br>Ate an extra meal (if woman is pregnant) ………………………..**2**<br>Ate 2 extra meals (if lactating mother)……………………………..**3**<br>Had an institutional delivery……………………………………………..**4**<br>Exclusively breastfed the baby for first six months……………**5**<br>Started complementary feeding from 6th month and continued till the 24th month……………………………………………………………….**6**
**2.6**	If you have not tested the behavior/practice, why not?<br>**if not tested skip to 2.9**	Did not think important….1<br>Lack of opportunity……2<br>Thought it was too complex….3<br>Did not understand the information well….4<br>Resource constraints….5<br>Lack of family support…6<br>Other, explain…7
**2.7**	Which of the practices did you adopt consistently	Haven't adopted…..**1**<br><br>Ate an extra meal (if woman is pregnant) ………………………..**2**<br>Ate 2 extra meals (if lactating mother)……………………………..**3**<br>Had an institutional delivery (for other deliveries)……………**4**<br>Exclusively breastfed the baby for first six months……………**5**<br>Started complementary feeding from 6th month and continued till the 24th month

**2.8**	If you have not adopted the behavior/practice, why not?	Did not think important….1<br>Lack of opportunity……2<br>Thought it was too complex….3<br>Did not understand the information well….4<br>Resource constraints….5<br>Lack of family support…6<br>Other, explain…7	
**2.9**	Have you shared the messages you heard about 1000 day practices with any one	Yes…..1, **if yes, skip to 2.11**<br>NO…..2	
**2.10**	If no, why? (note the qualitative response in the box)	Did not think important….1<br>Lack of opportunity……2<br>Thought it was too complex….3<br>Did not understand the information well….4<br>Did not think people will adopt….5<br>Other, explain…6	
**2.11**	What specific practices did you recommend? And to whom (note the details of the person to follow-up with)	Pregnant woman should eat an extra meal a day............................**1**<br>Breastfeeding mothers should eat 2 extra meals a day…………………**2**<br>Institutional delivery...**3**<br>Initiate breastfeeding during first hour of birth…………………………..**4**<br>Exclusive breastfeeding in the first 6 months………………………………**5**<br><br>Introduction of Complementary feeding along with breastfeeding from 6th month to the 24th months……………………………………………**6**	**WHOM and relationship: (for follow up)**

Video No. 3- Importance of Iron Folic tablet

S.NO	Question	Response
**3**	Has anyone in your community shared information on the **importance of iron folic tablet** with you in the last 3 months?	**Yes…1**<br>**No…2 If NO SKIP to 4**
**3.1**	Who /what is the source of this information?	Index SHG member…**1**<br>AWW…**2**<br>AHSA….**3**<br>ANM…**4**<br>Doctor…..**5**<br>Others within the HH: **6**<br>Others outside the HH<br>(if not index SHG member)…**7**<br>Radio….**8**<br>TV……**9**<br>Newspaper…..**10**<br>Others, specify:_______
**3.2**	What specific message about iron folic tablet did [Name of SHG member] share with you in the last 3 months? And how many times	There is a government scheme providing IFA tablets to pregnant women and adolescents……………………**1 Times** ☐<br>IFA helps in preventing anemia…………………………**2 Times** ☐<br>Anemia can cause small/low birth weight babies or premature birth…………………………………………………**3Times** ☐<br>Pregnant women should take IFA tablets starting their second trimester…………………………………………………**4 Times** ☐<br>Adolescent girls should take from 10-19years…….**5 Times** ☐

**3.3**	What did [NAME] convey about **preventing the side effects of taking IFA tablets**<br><br>*Check all that apply*	Take IFA tablets after the evening meal (not on an empty stomach)……………………………………………………………………………**1**<br>Drink extra water…………………………………………………………………**2**<br>Eat good amount of green leafy vegetables and fruits………..**3**
**3.4**	What did [NAME] convey about **the benefit of taking IFA tablets**<br><br>*Check all that apply*	Mother will be stronger and healthier during pregnancy……………………………………………………………………..**1**<br>Mother will be stronger and healthier during birth………**2**<br>Mother will give birth to a healthy baby and not premature delivery ………………………………………………………………………**3**<br>Mother will give birth to a healthy baby and not too small/low birth weight……………………………………………….**4**<br>**OTHER CODES…………………………………………………………..5**
**3.5**	Did you completely understand the message when it was shared to you?	Yes, completely understood the entire message/practice that was shared…..1<br>2-No, did not understand first but asked questions which the informant answered……2<br>3-No, did not understand even after asking questions because informant could not answer……3<br>4-No, did not understand and did not ask questions…4
**3.6**	Have you tested any of the recommended behaviors that were shared to you?<br>**If respondent not a pregnant woman/ adolescent girl/relevant case, skip to 3.8**<br>**If tested, skip to 3.8**	Haven't tested…..**1**<br><br>Have taken IFA tablets……**2**

**3.7**	If you have not tested the behavior/practice, why not?<br>**if not tested, skip to 3.10**	Did not think important….1<br>Lack of opportunity……2<br>Thought it was too complex….3<br>Did not understand the information well….4<br>Resource constraints….5<br>Lack of family support…6<br>Other, explain…7
**3.8**	Which of the practices did you adopt consistently	Haven't adopted…..**1**<br><br>Have adopted the practice of taking IFA tablets…..**2**
**3.9**	If you have not adopted the behavior/practice, why not?	Did not think important….1<br>Lack of opportunity……2<br>Thought it was too complex….3<br>Did not understand the information well….4<br>Resource constraints….5<br>Lack of family support…6<br>Other, explain…7
**3.10**	Have you shared the messages you heard about hand washing practices with any one	Yes…..1, **if yes, skip to 3.12**<br>NO…..2

**3.11**	If no, why? (note the qualitative response in the box)	Did not think important….1<br>Lack of opportunity……2<br>Thought it was too complex….3<br>Did not understand the information well….4<br>Did not think people will adopt….5<br>Other, explain…6	
**3.12**	What specific practices did you recommend? And to whom (note the details of the person to follow-up with)	There is a government scheme providing IFA tablets to pregnant women and adolescents……………………………………**1**<br>IFA helps in preventing anemia…………………………………………**2**<br>Anemia can cause small/low birth weight babies or premature birth…………………………………………………………………**3**<br>Pregnant women should take IFA tablets starting their second trimester………………………………………………………………**4**<br>Adolescent girls should take from 10-19years…………………**5**	**WHOM and relationship:**<br>**(for follow up)**

Video No.4 – Maternal Diet and Food Taboos

S.NO	Question	Response
**4**	Has anyone in your community shared information on the importance of **maternal diet and about food taboos** with you in the last 3 months?	**Yes…1**<br>**No…2 If NO SKIP to 5**
**4.1**	Who /what is the source of this information?	Index SHG member…**1**<br>AWW…**2**<br>AHSA….**3**<br>ANM…**4**<br>Doctor…..**5**<br>Others within the HH: **6**<br>Others outside the HH<br>(if not index SHG member)…**7**<br>Radio….**8**<br>TV……**9**<br>Newspaper…..**10**<br>Others, specify:_______
**4.2**	What specific message about maternal diet and food taboos did [Name of SHG member] share with you in the last 3 months? And how many times	Eat one additional meal during pregnancy and two additional meals during breastfeeding……………………………..**1** **Times** ☐<br>Eat green leafy vegetables, seasonal fruits and also animal source foods (2-3 times a week) ……………………….**2** **Times** ☐<br>Not eating adequately during pregnancy will lead to premature birth/ low birth weight Babies…………………………………………..**3** **Times** ☐

**4.3**	What did [NAME] convey about **the importance of maternal diet or about food taboos**<br>*Check all that apply*	Prevents premature birth and low birth weight babies**…1**<br>**OTHER CODES…………….2**
**4.4**	Did you completely understand the message when it was shared to you?	Yes, completely understood the entire message/practice that was shared…..1<br>2-No, did not understand first but asked questions which the informant answered……2<br>3-No, did not understand even after asking questions because informant could not answer……3<br>4-No, did not understand and did not ask questions…4
**4.5**	Have you tested any of the recommended behaviors that were shared to you?<br>**If respondent not a pregnant woman/relevant case, skip to 4.9**<br>**If tested, skip to 4.7**	Haven't tested…..**1**<br><br>Ate one additional meal during pregnancy and two additional meals during breastfeeding…………………………………………….…..**2**<br>Ate green leafy vegetables, seasonal fruits and also animal source foods (2-3 times a week) ……………………………………….**3**
**4.6**	If you have not tested the behavior/practice, why not?<br>**if not tested, skip to 4.9**	Did not think important….1<br>Lack of opportunity……2<br>Thought it was too complex….3<br>Did not understand the information well….4<br>Resource constraints….5<br>Lack of family support…6<br>Other, explain…7

**4.7**	Which of the practices did you adopt consistently	Haven't adopted…..**1**<br><br>Ate one additional meal during pregnancy and two additional meals during breastfeeding…………………………………………………**2**<br>Ate green leafy vegetables, seasonal fruits and also animal source foods (2-3 times a week) ………………………………………..**3**
**4.8**	If you have not adopted the behavior/practice, why not?	Did not think important….1<br>Lack of opportunity……2<br>Thought it was too complex….3<br>Did not understand the information well….4<br>Resource constraints….5<br>Lack of family support…6<br>Other, explain…7
**4.9**	Have you shared the messages you heard about maternal diet and food taboos with any one	Yes…..1, **if yes, skip to 4.11**<br>NO…..2
**4.10**	If no, why? (note the qualitative response in the box)	Did not think important….1<br>Lack of opportunity……2<br>Thought it was too complex….3<br>Did not understand the information well….4<br>Did not think people will adopt….5<br>Other, explain…6

**4.9**	What specific practices did you recommend? And to whom (note the details of the person to follow-up with)	Eat one additional meal during pregnancy and two additional meals during breastfeeding……………………….**1**<br>Eat green leafy vegetables, seasonal fruits and also animal source foods (2-3 times a week) ……………..**2**<br>Not eating adequately during pregnancy will lead to premature birth/ low birth weight babies...**3**	**WHOM and relationship:**<br>**(for follow up)**

Video No. 5- Maternal Workload during Pregnancy

S.NO	Question	Response
**5**	Has anyone in your community shared information on maternal workload during pregnancywith you in the last 3 months?	**Yes…1**<br>**No…2 If NO SKIP to 6**
**5.1**	Who /what is the source of this information?	Index SHG member…**1**<br>AWW…**2**<br>AHSA….**3**<br>ANM…**4**<br>Doctor…..**5**<br>Others within the HH: **6**<br>Others outside the HH<br>(if not index SHG member)…**7**<br>Radio….**8**<br>TV……**9**<br>Newspaper…..**10**<br>Others, specify:______
**5.2**	What specific message about maternal workload during pregnancy did [Name of SHG member] share with you in the last 3 months? And how many times	The need for more rest in the third trimester of pregnancy…….**1 Times** ☐<br>Strenuous work in the third trimester can lead to premature birth or low birth weight Babies…………………………………………………………………………………**2 Times** ☐<br>Family members should support pregnant women by helping with her chores and give more time to rest……………………………………………………………………………….**3 Times** ☐
**5.3**	What did [NAME] convey about **the benefit of less workload during third trimester of pregnancy?**<br>*Check all that apply*	Prevents premature birth and low birth weight babies**…1**<br>**OTHER CODES……………………….2**

**5.4**	Did you completely understand the message when it was shared to you?	Yes, completely understood the entire message/practice that was shared…..1<br>2-No, did not understand first but asked questions which the informant answered……2<br>3-No, did not understand even after asking questions because informant could not answer……3<br>4-No, did not understand and did not ask questions…4
**5.5**	Have you tested any of the recommended behaviors that were shared to you?<br>**If respondent not a pregnant woman/relevant case, skip to 5.9**<br>**If tested skip to 5.7**	Haven't tested…..**1**,<br><br>Takes more rest in the third trimester….**2**
**5.6**	If you have not tested the behavior/practice, why not?<br>**if not tested skip to 5.9**	Did not think important….1<br>Lack of opportunity……2<br>Thought it was too complex….3<br>Did not understand the information well….4<br>Resource constraints….5<br>Lack of family support…6<br>Other, explain…7
**5.7**	Which of the practices did you adopt consistently	Haven't adopted…..**1**<br><br>Takes more rest in the third trimester….**2**

**5.8**	If you have not adopted the behavior/practice, why not?	Did not think important….1<br>Lack of opportunity……2<br>Thought it was too complex….3<br>Did not understand the information well….4<br>Resource constraints….5<br>Lack of family support…6<br>Other, explain…7	
**5.9**	Have you shared the messages you heard about maternal workload during pregnancy with any one	Yes…..1, **if yes, skip to 5.11**<br>NO…..2	
**5.10**	If no, why? (note the qualitative response in the box)	Did not think important….1<br>Lack of opportunity……2<br>Thought it was too complex….3<br>Did not understand the information well….4<br>Did not think people will adopt….5<br>Other, explain…6	
**5.9**	What specific practices did you recommend? And to whom (note the details of the person to follow-up with)	The need for more rest in the third trimester of pregnancy…….**1**<br>Strenuous work in the third trimester can lead to premature birth or low birth weight babies …**2**<br>Family members should support pregnant women by helping with her chores and give more time to rest...**3**	**WHOM and relationship:**<br>**(for follow up)**

Video No. 6- Importance of Exclusive Breastfeeding

S.NO	Question	Response
**6**	Has anyone in your community shared information on the **importance of exclusive breast feeding** with you in the last 3 months?	**Yes…1**<br>**No…2 If NO SKIP to 7**
**6.1**	Who /what is the source of this information?	Index SHG member…**1**<br>AWW…**2**<br>AHSA….**3**<br>ANM…**4**<br>Doctor…..**5**<br>Others within the HH: **6**<br>Others outside the HH<br>(if not index SHG member)…**7**<br>Radio….**8**<br>TV……**9**<br>Newspaper…..**10**<br>Others, specify:_______
**6.2**	What specific message about exclusive breastfeeding did [Name of SHG member] share with you in the last 3 months? And how many times	Babies should be put to the breast within one hour after birth……………………………………………………………………**1 Times** ☐<br>Babies should be given only breast milk until 6 months and should not be given honey, or any other liquids, not even water…………………………………………………………………**2Times** ☐<br>Mothers should breastfeed frequently, 'on demand', both day and night……………………………………………………………**3 Times** ☐

**6.3**	Did you completely understand the message when it was shared to you?	Yes, completely understood the entire message/practice that was shared…..1<br>2-No, did not understand first but asked questions which the informant answered……2<br>3-No, did not understand even after asking questions because informant could not answer……3<br>4-No, did not understand and did not ask questions…4
**6.4**	Have you tested any of the recommended behaviors that were shared to you?<br>**If respondent not a lactating mother/relevant case, skip to 6.8**<br>**If tested, skip to 6.6**	Haven't tested…..**1**<br><br>Initiated breastfeeding in the first hour of birth…..**2**<br><br>Exclusively breastfed the baby for first 6 months…**3**
**6.5**	If you have not tested the behavior/practice, why not?<br>**if not tested skip to 6.8**	Did not think important….1<br>Lack of opportunity……2<br>Thought it was too complex….3<br>Did not understand the information well….4<br>Resource constraints….5<br>Lack of family support…6<br>Other, explain…7
**6.6**	Which of the practices did you adopt consistently	Haven't adopted…..**1**<br>Initiated breastfeeding in the first hour of birth…..**2**<br><br>Exclusively breastfed the baby for first 6 months…**3**

**6.7**	If you have not tested the behavior/practice, why not?	Did not think important….1<br>Lack of opportunity……2<br>Thought it was too complex….3<br>Did not understand the information well….4<br>Resource constraints….5<br>Lack of family support…6<br>Other, explain…7	
**6.8**	Have you shared the messages you heard about hand washing practices with any one	Yes…..1, **if yes, skip to 6.10**<br>NO…..2	
**6.9**	If no, why? (note the qualitative response in the box)	Did not think important….1<br>Lack of opportunity……2<br>Thought it was too complex….3<br>Did not understand the information well….4<br>Did not think people will adopt….5<br>Other, explain…6	
**6.10**	What specific practices did you recommend? And to whom (note the details of the person to follow-up with)	Babies should be put to the breast within one hour after birth………………………………………………………………………**1**<br><br>Babies should be given only breast milk until 6 months and should not be given honey, or any other liquids, not even water…………………………………………………………………..**2**<br>Mothers should breastfeed frequently, 'on demand', both day and night………………………………………………………….......**3**	**WHOM and relationship:**<br>**(for follow up)**

Video No. 7: Managing exclusive breastfeeding by working mother

S.NO	Question	Response
**7**	Has anyone in your community shared information on **managing exclusive breast feeding by working mothers** with you in the last 3 months?	**Yes…1**<br>**No…2 If NO SKIP to 8**
**7.1**	Who /what is the source of this information?	Index SHG member…**1**<br>AWW…**2**<br>AHSA….**3**<br>ANM…**4**<br>Doctor…..**5**<br>Others within the HH: **6**<br>Others outside the HH<br>(if not index SHG member)…**7**<br>Radio….**8**<br>TV……**9**<br>Newspaper…..**10**<br>Others, specify:_______
**7.2**	What specific message about managing exclusive breastfeeding by working mothers did [Name of SHG member] share with you in the last 3 months? And how many times	Working mothers should breastfeed the baby more times in the morning and night, for longer durations, when with the baby……………………**1 Times**☐<br><br>Families can support working mothers so that they can exclusively breastfeed their babies until 6 months and continue to breastfeed for up to two years or more…………………………………………………………………..**2Times**☐<br>Working mothers should express breast milk for the baby……………………………………………………………………...**3 Times**☐

**7.3**	When [NAME] shared information with you about **how to family can support working mothers to exclusively breastfeed** what specific practices were mentioned<br><br>*Check all that apply*	Family members can share in the mother's workload in house and outside house also…………………………………………………………**1**<br>A family member can bring the baby to the mother to breastfeed in the field (or other workplace)……………………………**2**<br>A family member can accompany the mother to the field during the day to help take care of the baby while the mother works……………………………………………………………………………………**3**
**7.4**	Did you completely understand the message when it was shared to you?	Yes, completely understood the entire message/practice that was shared…..1<br>2-No, did not understand first but asked questions which the informant answered……2<br>3-No, did not understand even after asking questions because informant could not answer……3<br>4-No, did not understand and did not ask questions…4
**7.5**	Have you tested any of the recommended behaviors that were shared to you?<br>**If respondent not a working mother with a baby less than 6months old/relevant case, skip to 7.9**<br>**If tested, skip to 7.7**	Haven't tested…..**1**<br><br>Fed baby for longer hours in the morning and night for more number of times……………………………………………………………..**2**<br><br>Family member brought baby to workplace to be fed…………**3**<br><br>Expressed breast milk and caregiver fed baby……………………..**4**
**7.6**	If you have not tested the behavior/practice, why not?<br>**if not tested skip to 7.9**	Did not think important….1<br>Lack of opportunity……2<br>Thought it was too complex….3<br>Did not understand the information well….4<br>Resource constraints….5<br>Lack of family support…6<br>Other, explain…7

**7.7**	Which of the practices did you adopt consistently	Haven't adopted…..**1**<br><br>Fed baby for longer hours in the morning and night for more number of times…………………………………………………………………..**2**<br><br>Family member brought baby to workplace to be fed…………**3**<br><br>Expressed breast milk and caregiver fed baby……………………..**4**
**7.8**	If you have not adopted the behavior/practice, why not?	Did not think important….1<br>Lack of opportunity……2<br>Thought it was too complex….3<br>Did not understand the information well….4<br>Resource constraints….5<br>Lack of family support…6<br>Other, explain…7
**7.9**	Have you shared the messages you heard about hand washing practices with any one	Yes…..1, **if yes, skip to 7.11**<br>NO…..2
**7.10**	If no, why? (note the qualitative response in the box)	Did not think important….1<br>Lack of opportunity……2<br>Thought it was too complex….3<br>Did not understand the information well….4<br>Did not think people will adopt….5<br>Other, explain…6

**7.11**	What specific practices did you recommend? And to whom (note the details of the person to follow-up with)	Babies should be put to the breast within one hour after birth…………………………………………………………………….**1**<br><br>Babies should be given only breast milk until 6 months and should not be given honey, or any other liquids, not even water...**2**<br>Mothers should breastfeed frequently, 'on demand', both day and night…………………………………………….................**3**	**WHOM and relationship:**<br>**(for follow up)**
