# Supplementary material for: Adapting Agriculture Platforms for Nutrition: A Case Study of a Participatory, Video-Based Agricultural Extension Platform in India
Source: PLoS One. 2016 Oct 13;11(10):e0164002. doi: 10.1371/journal.pone.0164002 (PMC5063370; doi:10.1371/journal.pone.0164002)
Supplement: S9 File — (PDF) [file pone.0164002.s009.pdf]

Digital Green Feasibility Study: Structured Observation

Instructions for the observer:

This guide will serve as a reference for approaching the structured observation during video dissemination activities in villages included in the sample. This observation should not intrude or disrupt the regular proceedings of the dissemination, but rather the observer should take pains to be unobtrusive as to not influence the activities at hand. It is of utmost importance for the observer to pay attention to detail while using the guide, but also to seize upon opportunities to gather any interesting data that may fall outside the parameters of the observation guide template.

<u>*Observation Guide*</u>

1. Local Context

Appropriateness of space: Examine the physical and social characteristics of the space in which the dissemination is being held.

How many people attended the video session?	☐☐
Are there community members other than SHG members who attended the video?	No
	Yes
	If Yes, who are they? Husbands? Non-SHG mothers?
Did the behavior of these non-SHG community members affect the dissemination in any way?	No
	Yes
	If Yes, how?
Were there any children in attendance for the dissemination?	No
	Yes
Did the behavior of the children affect the dissemination in any way?	No
	Yes
	If Yes, how?

Is the space of sufficient size for the number of attendees?<br>(How crowded was the room where the sessions were conducted)	No, it was crowded
	Yes. It was sufficient space and not crowded
Was the room darkened during screening?	No
	Yes
Was there enough light during discussion before and after the screening?	No
	Yes

2. **Condition of the Equipment**

What was the condition of equipment used for the session?	All equipment in working condition
	Minimal malfunction in equipment
	Significant malfunction in the equipment
	Some equipment not transferred

3. **Behavior of CSPs**:

Closely observe the behavior and actions of the CSP during the video dissemination session.

a. Introduction

How was the introduction & initial discussion?	Introduced external attendees & name of video
	Made partial introductions
	Started screening without any introductions

Sharing the purpose of screening a video on the specific topic	Shares clearly the purpose of screening the video
	Shares purpose, but not clearly
	Does not share the purpose at all

b. *Technical ability*:

Was the equipment set up and connected in an error free manner?	Set up, connected without errors
	Set up, connected some errors
	Unsuccessful usage of equipment
Any other technical issue faced during dissemination?	No
	Yes
	List issue, if Yes

c. *Interaction with community*:

Number of adopters of previous video	☐☐
Number of adopter experience sharing	☐☐

How was the feedback on experiences/adoptions of last video invited and shared?	Feedback invited and shared by all adopters
	Feedback invited and shared by some adopters
	Feedback invited but not shared
	Feedback not invited

d. Knowledge of Material and Facilitation skills

Initiating discussions and encourages participation	Initiates and encourages discussions and participation
	Needs some improvement
	Does not initiate or encourage discussions and participation
Initiate relevant questions beyond video prompts	Initiates questions beyond prompts and responses received
	Initiates questions beyond prompts but no responses received
	Does not initiate or encourage questions beyond prompts
Encourages silent participants to speak	Encourages all silent participants to speak
	Encourages some silent participants to speak
	Does not encourage anyone
Helping generate ideas/options to enable adoption of practice	Helps generate ideas and options to enable adoption
	Needs some improvement
	Does not help in generating ideas/options to enable adoption
Appropriate responses to questions	Well Informed and clear response
	Needs some improvement
	Does not know answers to questions
Summarizing at the end of each discussion point	Clear and concise summary
	Needs some improvement
	Unclear summary
Summarizing at the end of dissemination	Clear and concise summary
	Needs some improvement
	Unclear summary

4. **Community reception of video dissemination***:*

a. Engagement:

Do SHG members readily participate in the discussions- are they comfortable asking questions?	No, they hesitate to participate and ask no questions
	The ask few questions and participate when prompted; can improve
	They need no prompts and readily take part and ask questions

**5. Any other observations**
